# Dominant Bacterial Phyla from the Human Gut Show Widespread Ability To Transform and Conjugate Bile Acids

**DOI:** 10.1128/msystems.00805-21

**Published:** 2021-08-31

**Authors:** L. N. Lucas, K. Barrett, R. L. Kerby, Q. Zhang, L. E. Cattaneo, D. Stevenson, F. E. Rey, D. Amador-Noguez

**Affiliations:** a Department of Bacteriology, University of Wisconsin—Madison, Madison, Wisconsin, USA; b Microbiology Doctoral Training Program, University of Wisconsin—Madison, Madison, Wisconsin, USA; Vall d'Hebron Research Institute (Ed. Mediterranea)

**Keywords:** bile acids, gut bacteria, microbially conjugated bile acids, conjugation, mass spectrometry, microbiome

## Abstract

Gut bacteria influence human physiology by chemically modifying host-synthesized primary bile acids. These modified bile acids, known as secondary bile acids, can act as signaling molecules that modulate host lipid, glucose, and energy metabolism and affect gut microbiota composition via selective antimicrobial properties. However, knowledge regarding the bile acid-transforming capabilities of individual gut microbes remains limited. To help address this knowledge gap, we screened 72 bacterial isolates, spanning seven major phyla commonly found in the human gut, for their ability to chemically modify unconjugated bile acids. We found that 43 isolates, representing 41 species, were capable of *in vitro* modification of one or more of the three most abundant unconjugated bile acids in humans: cholic acid, chenodeoxycholic acid, and deoxycholic acid. Of these, 32 species have not been previously described as bile acid transformers. The most prevalent bile acid transformations detected were oxidation of 3α-, 7α-, or 12α-hydroxyl groups on the steroid core, a reaction catalyzed by hydroxysteroid dehydrogenases. In addition, we found 7α-dehydroxylation activity to be distributed across various bacterial genera, and we observed several other complex bile acid transformations. Finally, our screen revealed widespread bacterial conjugation of primary and secondary bile acids to glycine, a process that was thought to only occur in the liver, and to 15 other amino acids, resulting in the discovery of 44 novel microbially conjugated bile acids.

**IMPORTANCE** Our current knowledge regarding microbial bile acid transformations comes primarily from biochemical studies on a relatively small number of species or from bioinformatic predictions that rely on homology to known bile acid-transforming enzyme sequences. Therefore, much remains to be learned regarding the variety of bile acid transformations and their representation across gut microbial species. By carrying out a systematic investigation of bacterial species commonly found in the human intestinal tract, this study helps better define the gut bacteria that impact composition of the bile acid pool, which has implications in the context of metabolic disorders and cancers of the digestive tract. Our results greatly expand upon the list of bacterial species known to perform different types of bile acid transformations. This knowledge will be vital for assessing the causal connections between the microbiome, bile acid pool composition, and human health.

## INTRODUCTION

Within the last decade, the central role that the gut microbiota plays in human health has become widely recognized. A fundamental way by which the gut microbiota affects human physiology is via a wide variety of microbially encoded biochemical activities such as vitamin synthesis, production of small organic acids, and modification of bile acids (1). While each of these has a significant impact on human health, chemical modification of bile acids by gut microbes is of particular interest due to the fact that bacterially modified bile acids can act as signaling molecules (i.e., hormones) within the host (2–7) and can also directly modulate gut microbiota composition (4, 5, 8).

**FIG 1:**
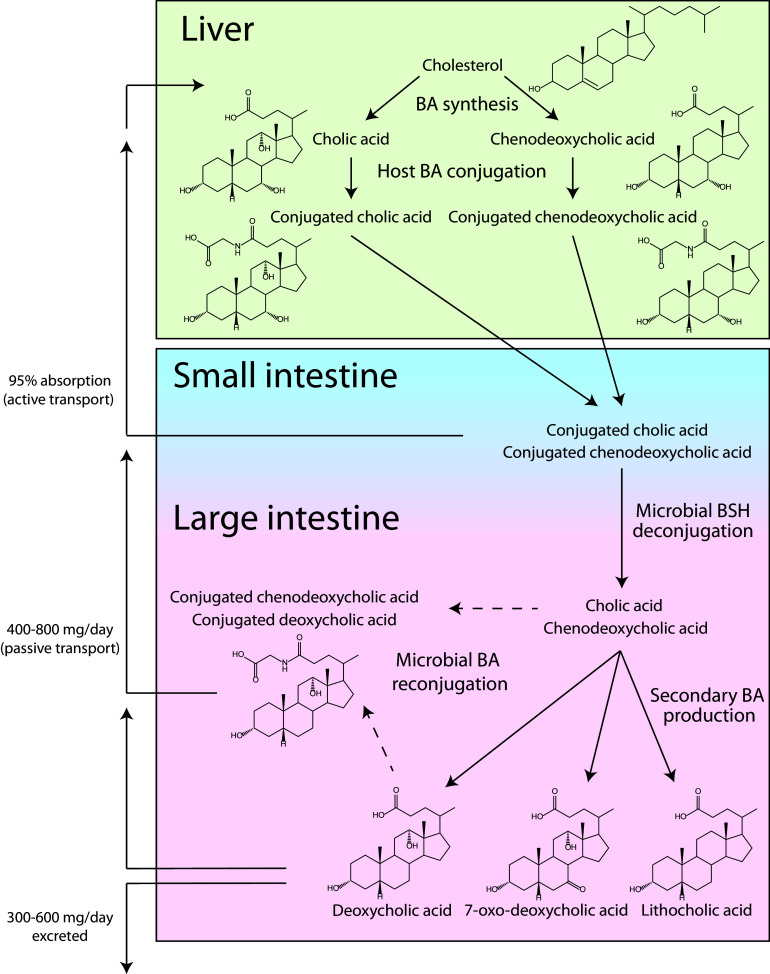
Bile acid production and enterohepatic circulation. Conjugated bile acids are synthesized from cholesterol in the liver and stored in bile in the gallbladder. After being released from the gallbladder into the duodenum, bile acids travel through the jejunum and into the ileum. Ninety-five percent of bile acids are actively absorbed from the small intestine and returned to the liver via enterohepatic circulation. The remaining 5% that reach the large intestine can be passively reabsorbed or deconjugated and transformed into secondary bile acids by the gut microbiota. Bacterial transformation of bile acids can also occur in the small intestine to a lesser extent. Secondary bile acids can be excreted, or they can be absorbed in the large intestine to join enterohepatic circulation. This figure shows primary bile acids conjugated to glycine moieties.

The human liver produces two bile acids, termed primary bile acids, from cholesterol: cholic acid (CA) and chenodeoxycholic acid (CDCA) (Fig. 1) (9). After synthesis, primary bile acids in the liver are conjugated to taurine or glycine, which enhances their solubility and facilitates their circulation within the body (10). Conjugated bile acids are then shuttled through the gallbladder into the small intestine, where they act as detergents to help emulsify and solubilize fats and facilitate the transport of lipids and lipid soluble vitamins (Fig. 1). As bile acids circulate, the majority (∼95%) are actively absorbed across the distal small intestine wall (Fig. 1) (8). The reabsorbed bile acids are returned to the liver and gallbladder and continue through this cycle, called enterohepatic circulation. However, a small amount (∼5%) escape ileal bile salt transport and enter the large intestine. In the large intestine primarily, but also in the small intestine, bile acids are subjected to chemical modification by bacteria (Fig. 1) (11). Primary bile acids that have been modified by bacteria are termed secondary bile acids; more than 50 secondary bile acids have so far been documented in human feces (12–15). Secondary bile acids are passively reabsorbed through the large intestine wall and into the bloodstream, where they join other bile acids undergoing enterohepatic circulation (11–14).

One of the most widespread bile acid transformations carried out by gut bacteria is deconjugation (i.e., removal of the glycine or taurine moieties) of glycine- and taurine-conjugated primary bile acids (Fig. 1) (2, 14, 16, 17). The hydrolysis of the amide bond connecting taurine or glycine to the bile acid steroid core is performed by bacterial bile salt hydrolases (BSH) (10, 18, 19). BSH activity appears to be common in gut microbes, though less widespread in Gram-negative than in Gram-positive bacteria (11, 16, 20–23). The efficiency by which bile acids are recirculated throughout the body is dependent on their structure; glycine- and taurine-conjugated bile acids are more readily removed from the gastrointestinal (GI) tract by active transport and are also more readily taken up by the liver during systemic circulation (2, 5, 24, 25). In humans, CA, CDCA, deoxycholic acid (DCA), ursodeoxycholic acid (UDCA), and lithocholic acid (LCA) can be reconjugated to glycine or taurine by host enzymes in the liver (25).

**FIG 2:**
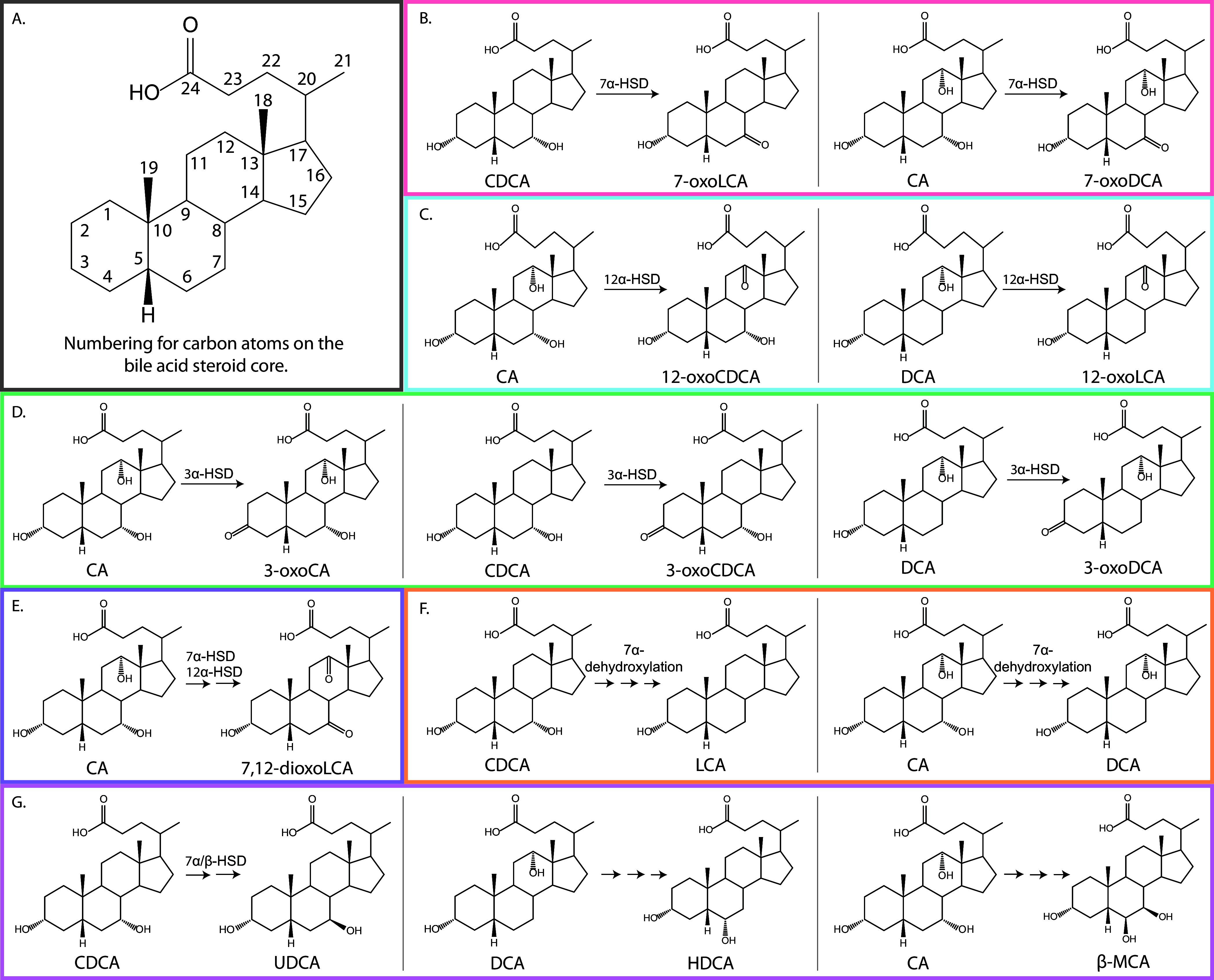
Secondary bile acid production observed *in vitro*. (A) Numbering of carbon atoms on the bile acid steroid core. (B) 7α-dehydrogenation of CDCA and CA. (C) 12α-dehydrogenation of CA and DCA. (D) 3α-dehydrogenation of CA, CDCA, and DCA. (E) Transformation of CA into 7,12-dioxoLCA. (F) 7α-dehydroxylation of CDCA and CA. (G) Other transformations. CA, cholic acid; CDCA, chenodeoxycholic acid; DCA, deoxycholic acid; HDCA, hyodeoxycholic acid; LCA, lithocholic acid; UDCA, ursodeoxycholic acid; β-MCA, β-muricholic acid; 3-oxoCA, 3-oxocholic acid; 3-oxoCDCA, 3-oxochenodeoxycholic acid; 3-oxoDCA, 3-oxodeoxycholic acid; 7-oxoDCA, 7-oxodeoxycholic acid; 7-oxoLCA, 7-oxolithocholic acid; 12-oxoCDCA, 12-oxochenodeoxycholic acid; 12-oxoLCA, 12-oxolithocholic acid; 7,12-dioxoLCA, 7,12-dioxolithocholic acid. A complete list of bile acids and abbreviations used in this study can be found in Table S2.

Following deconjugation, bacterial production of secondary bile acids can involve several categories of reactions, including dehydroxylation, dehydrogenation, esterification, epimerization, and oxidation (9). These chemical transformations may be carried out by a single species harboring multiple enzymes or may require the sequential action of different bacterial species, each possessing distinct capabilities (11). One of the most common enzyme classes in bile acid transformations is hydroxysteroid dehydrogenases (HSDs) (26, 27). HSDs catalyze the reversible oxidation of hydroxyl groups on the C-3, C-7, and C-12 carbon positions of the bile acid steroid core (Fig. 2A to E). HSDs have stereospecificity; for example, a 3α-HSD can oxidize an α-OH group at the C-3 position, whereas a 3β-HSD can oxidize a β-OH group at the C-3 carbon. Thus, the sequential action by both α- and β-HSDs can result in epimerization of an -OH group on the bile acid steroid core (28–30). Another well-recognized transformation is the dehydroxylation of the primary bile acids CA and CDCA at the α-oriented hydroxyl group on C-7, which results in the two most common secondary bile acid species, DCA and LCA (Fig. 2F) (30).

10.1128/mSystems.00805-21.6 TABLE S2 Bile acid names, retention times, and structure description for standards used for LC-MS/MS analysis. Download Table S2, DOCX file, 0.1 MB. Copyright © 2021 Lucas et al. 2021 Lucas et al. https://creativecommons.org/licenses/by/4.0/ This content is distributed under the terms of the Creative Commons Attribution 4.0 International license.

Variations in gut microbial community makeup can lead to variable composition of an individual’s bile acid pool (5, 12, 33, 34), with differential effects on both the host and gut microbiota. Bile acids can bind and modulate the activity of nuclear hormone receptors (NHRs) in the liver, including farnesoid X receptor (FXR), vitamin D receptor (VDR), and the pregnane-activated receptor (PXR), which are involved in the regulation bile acid homeostasis, xenobiotic metabolism, triglyceride metabolism, and glucose metabolism (2–4, 7). Bile acids can also modulate activity of G protein-coupled receptors (GPCRs) in the intestine, which participate in the regulation of bile acid metabolism, energy expenditure, and glucose metabolism (2–4, 6). By interacting with these receptors in the liver and intestine, bile acids can elicit broad effects on host physiology (1–8). Deviations from typical bile acid levels have been associated with illnesses such as cholesterol gallstone disease (35), recurrent GI tract bacterial infections (36), and liver and colon cancers (37, 38). There is growing evidence that bile acids produced by HSD activity are involved in the pathogeneses of hormone-dependent cancer, hypertension, and obesity (26), and high levels of hyodeoxycholic acid (HDCA), a ligand for liver X receptors and GPCRs, have been associated with cholestatic liver disease or intestinal malabsorption (39, 40). In contrast to the association of bile acids with disease, UDCA is used as a therapeutic for cholesterol gallstones, primary biliary cirrhosis, primary sclerosing cholangitis, and recurrent pancreatitis (27).

The full variety of bile acid transformations and their representation across gut microbial species are still unknown; current knowledge is primarily based on biochemical studies from a relatively small number of species or bioinformatic searches that rely on known sequences of genes involved in bile acid transformation to make predictions (18, 28, 35, 41–47). Because of this, our current knowledge regarding microbial bile acid transformations is not sufficient to predict how a particular microbe impacts the bile acid pool and, thus, host physiology. To shed light on these questions, we performed a systematic *in vitro* investigation of the bile acid-transforming capabilities of 72 bacterial species commonly found in the human intestinal tract. Our results significantly expand upon the lists of bacterial species known to perform bile acid transformations, and we also identify novel conjugated secondary bile acids. The results of this study will help scientists better understand how the gut microbiome shapes the composition of the bile acid pool.

## RESULTS AND DISCUSSION

### *In vitro* screen for bile acid-transforming activity in gut bacteria.

Seventy-two bacterial isolates, representing 70 species across seven phyla found in the human gut, were assessed for their *in vitro* ability to chemically transform the primary bile acids cholic acid (CA) and chenodeoxycholic acid (CDCA) and the prominent secondary bile acid deoxycholic acid (DCA) (Table S1). All strains used in this study, with the exception of Clostridium scindens, were previously sequenced, and their genomes are publicly available (Table S1). The *C. scindens* strain was newly isolated from a human fecal sample (see Materials and Methods). Bacterial isolates were incubated with each of these three bile acids separately (i.e., CA, DCA, and CDCA) at 100 μM concentrations, which are within physiological ranges (48, 49). In addition, we incubated each isolate with all three bile acids combined, totaling 300 μM. Samples were collected at 24 and 48 h post-inoculation for quantitation of bile acids (see Materials and Methods). We used a non-targeted high-pressure liquid chromatography-tandem mass spectrometry (HPLC-MS/MS) method to screen for the presence of transformed bile acids (39 of which were directly matched to standards), including conjugated bile acids (see Table S2).

10.1128/mSystems.00805-21.5 TABLE S1 Bacterial strain names, identification (ID) number, and accession numbers. Download Table S1, DOCX file, 0.033 MB. Copyright © 2021 Lucas et al. 2021 Lucas et al. https://creativecommons.org/licenses/by/4.0/ This content is distributed under the terms of the Creative Commons Attribution 4.0 International license.

**FIG 3:**
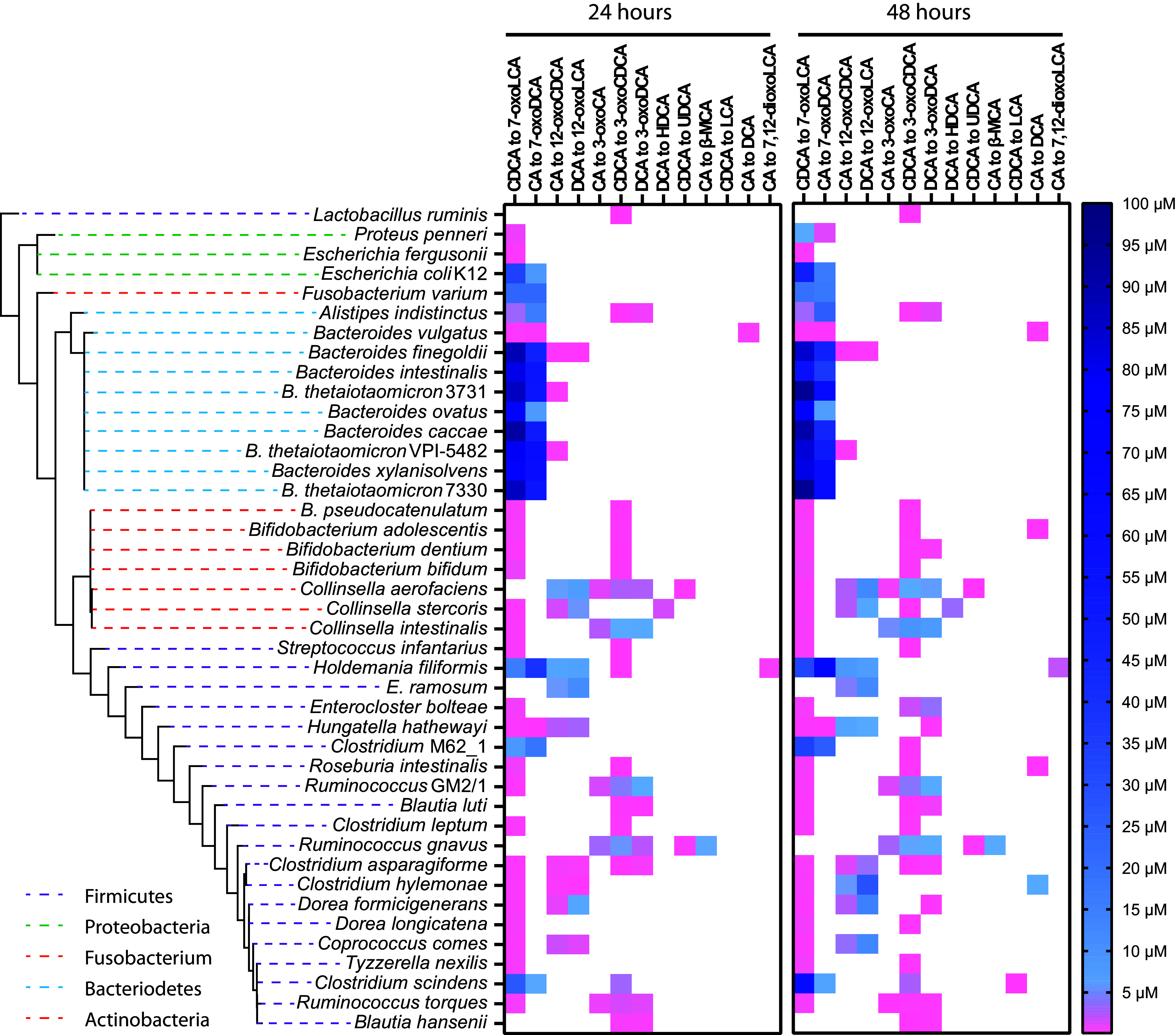
Secondary bile acid production across bacterial strains. The heat map shows the bile acid steroid core transformations carried out by each isolate. The color scale denotes amounts of secondary bile acid produced at 24 (left) and 48 h (right). Bacteria were provided with 100 μM cholic acid (CA), chenodeoxycholic acid (CDCA), or deoxycholic acid (DCA). The detection limit was below 0.05 μM for all bile acids. Phyla information is indicated by color-coded dashed lines in the phylogenetic tree. Heat maps for bile acid production when CA, CDCA, and DCA were added in combination are shown in Fig. S2.

We observed that bile acid-transforming activity was highly prevalent among the gut microbes analyzed and was not phylogenetically constrained (Fig. S1). Of the 72 strains screened, we found that 43 (representing 41 species) performed at least one bile acid modification on one or more of the bile acids tested. Of the 43 strains that showed bile acid-transforming capabilities, 32 species were novel bile acid transformers. Additionally, of the 9 species already known to transform bile acids, all showed additional capabilities not previously recognized. The 43 bacterial strains exhibited a range of bile acid transformations: 42 possessed hydroxysteroid dehydrogenase (HSD) activity, 5 showed 7α-dehydroxylation activity, and 3 performed other less-well-studied transformations on the bile acid core (Fig. 2, 3, and S1). Interestingly, we also found that 28 strains, representing 27 species, were capable of conjugating bile acids to amino acids. Most strains with bile acid-transforming capabilities were found in the *Actinobacteria*, *Firmicutes*, or *Bacteroidetes* phyla, which were our most heavily sampled phyla.

10.1128/mSystems.00805-21.1 FIG S1 Phylogenetic tree transformation summary of assessed bacterial isolates. Strains are listed along with the types of bile acid transformations that they can perform. For strains listed with conjugation abilities, all types of amino acid conjugation were grouped together, i.e., each marked strain can conjugate a bile acid to either glycine or other amino acids (Table S4). Previous bioinformatic predictions of bile acid transformation capabilities (26, 27) are also indicated. Phylum information is color coded on the tree. The tree was constructed using publicly available genomes, see Materials and Methods. Download FIG S1, PDF file, 0.8 MB. Copyright © 2021 Lucas et al. 2021 Lucas et al. https://creativecommons.org/licenses/by/4.0/ This content is distributed under the terms of the Creative Commons Attribution 4.0 International license.

10.1128/mSystems.00805-21.2 FIG S2 Bile acid transformations across bacterial strains. The heat map shows bile acid transformations carried out by each isolate. The color scale denotes amounts of secondary bile acid produced at 24 (left) and 48 h (right). Bacteria were provided with 100 μM cholic acid (CA), chenodeoxycholic acid (CDCA), and deoxycholic acid (DCA), totaling 300 μM combined bile acids. The detection limit was below 0.05 μM for all bile acids. DCA was one of the added bile acids; therefore, its production was not quantified in this experiment. Phyla information is indicated by the color-coded dashed lines in the phylogenetic tree on the left. Download FIG S2, PDF file, 0.08 MB. Copyright © 2021 Lucas et al. 2021 Lucas et al. https://creativecommons.org/licenses/by/4.0/ This content is distributed under the terms of the Creative Commons Attribution 4.0 International license.

Our *in vitro* analysis also revealed a wide range in the efficiency by which different bacterial isolates transform bile acids; for example, some microbes transformed >90% of the added bile acid, while others transformed 5% or less. Most bile acid transformations occurred within 24 h of growth, but several strains displayed a measurable increase in bile acid production from 24 to 48 h (Fig. 3 and S2). Finally, we observed no significant differences (*P* > 0.05, two-tailed paired *t* test) in bile acid transformations when bile acid substrates were added individually or in combination.

### 3α-, 7α-, and 12α-hydroxysteroid dehydrogenase activity.

The most common bile acid transformation that we encountered in our screen was oxidation of 3α-, 7α-, or 12α-hydroxyl groups on the steroid core (Fig. 2A to E, 3, S1, and S2). 7α-HSD activity was present in 38 strains and yielded the highest concentrations of secondary bile acids in our screen (Fig. 2B, 3, S1, and S2). On average, the strains that possessed 7α-HSD activity transformed ∼30.8 μM CA (ranging from 0.12 to 66.4 μM) and ∼22.8 μM CDCA (ranging from 0.16 to 93.9 μM) by 48 h (Fig. 3 and Table S3). Members of the *Bacteroidaceae* family, with the exception of Bacteroides vulgatus, exhibited the highest production of secondary bile acids, transforming 46.1 ± 18.5 μM or 82.4 ± 12.2 μM added CA or CDCA, respectively, into 7-oxo bile acid intermediates by 48 h (Fig. 3 and Table S3). Several strains of Bacteroides thetaiotaomicron have been shown to exhibit 7α-HSD activity, but the B. thetaiotaomicron 3731, VPI-5482, and 7330 strains, all of which displayed high bile acid-transforming activity, were not previously described (Fig. 3) (50). Additionally, we corroborated that Bacteroides intestinalis exhibited 7α-HSD activity on both CA and CDCA (44). Also consistent with previous findings, we found that Escherichia coli K-12 substrain MG1655 possessed 7α-HSD activity, acting on both CA and CDCA (9, 11, 44, 51–53).

10.1128/mSystems.00805-21.7 TABLE S3 Bile acid steroid core transformation data. Download Table S3, XLSX file, 0.1 MB. Copyright © 2021 Lucas et al. 2021 Lucas et al. https://creativecommons.org/licenses/by/4.0/ This content is distributed under the terms of the Creative Commons Attribution 4.0 International license.

**FIG 4:**
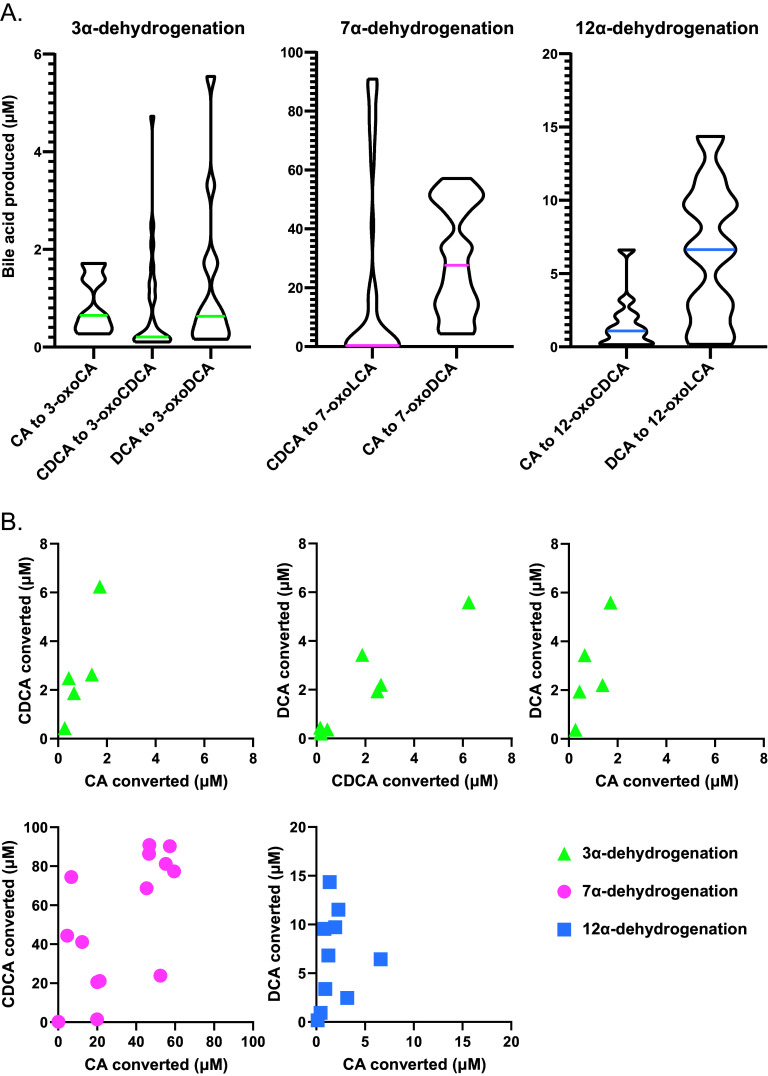
Relative activity and specificity of α-dehydrogenase activity. (A) 3α-, 7α-, and 12α-dehydrogenation activity when bile acids were administered separately. The median production of oxo bile acids is denoted by a solid colored line. Data shown are the average of 24 and 48 h measurements. (B) For each isolate capable of 3α-dehydrogenation, the amounts of CDCA, CA, and DCA transformed were plotted against each other. For each isolate capable of 7α-dehydrogenation, the amounts of CA and CDCA transformed were plotted against each other. For each isolate capable of 12α-dehydrogenation, the amounts of CA and DCA transformed were plotted against each other. Data shown are the averages of 24 and 48 h measurements.

We observed 3α-HSD activity in 25 isolates, 16 of which were *Firmicutes*, mostly in the *Lachnospiraceae* family (Fig. 2D, 3, and S2). On average, these strains displayed much lower activity than those possessing 7α-HSD activity (Fig. 4A). By 48 h, the average concentrations of secondary bile acids produced by 3α-HSD activity were ∼0.93 μM (ranging from 0.12 to 2.29 μM) for CA, ∼0.97 μM (ranging from 0.10 to 9.35 μM) for CDCA, and ∼1.54 μM (ranging from 0.16 to 7.23 μM) for DCA (Fig. 3 and Table S3). Most strains that exhibited 3α-HSD activity only acted on one or two of the bile acid substrates, but two of three *Collinsella* strains and all three *Ruminococcus* strains tested were able to transform all three added bile acids, CA, CDCA, and DCA (Fig. 3). Furthermore, these five strains produced the highest concentrations of 3-oxo bile acids (Fig. 3 and Table S3). Collinsella aerofaciens has only been shown to produce 3-oxoCA from CA; thus, production of 3-oxoDCA and 3-oxoCDCA expand upon its previously known bile acid-transforming capabilities (16). Our lab isolate of *C. scindens* produced 3-oxoCDCA from CDCA and 3-oxoCA from CA but did not produce 3-oxoDCA from DCA (Fig. 3 and S2), which is consistent with a previous study reporting that both *C. scindens* VPI 12708 and *C. scindens* ATCC 35704 produce 3-oxo bile acids from both CA and CDCA (45).

12α-HSD activity was detected in 12 strains (Fig. 2C, 3, S1, and S2). 12α-HSD activity was previously detected primarily in the genus *Clostridium*, but our results show it to be much more widespread (Fig. 3 and S1) (12, 27). Members of the *Firmicutes* and *Actinobacteria* phyla produced higher concentrations of 12-oxo bile acids than members of the *Bacteroidetes* (Fig. 3 and S1). On average, strains that exhibited 12α-HSD activity transformed ∼2.0 μM CA (ranging from 0.09 to 7.92 μM) and ∼9.74 μM DCA (ranging from 0.19 to 28.6 μM) at 48 h, a level of activity that is intermediate between the 7α-HSD and 3α-HSD activities discussed above (Fig. 3 and 4A; Table S3). In several instances, the 12-oxo bile acid concentration increased significantly between 24 and 48 h (*P* < 0.005, two-tailed paired *t* test) (Fig. 3 and S2).

12α-HSD activity on bile acids was demonstrated previously in 7α-dehydroxylating bacteria Clostridium leptum, *C. scindens*, and Clostridium hylemonae as well as in the gut commensal *C. aerofaciens* (16, 27). We observed that *C. hylemonae* produced 12-oxo bile acids from CA and DCA, but we did not observe 12α-HSD activity in *C. leptum* and *C. scindens* (Fig. 3) (11). Consistent with previous observations (16), we found that *C. aerofaciens* transformed CA and CDCA into 12-oxoCDCA and 12-oxoLCA, respectively.

Lastly, we found that Holdemania filiformis produced a small amount of 7, 12-dioxoLCA from CA by 48 h (Fig. 3 and S2, and Table S3). The transformation of CA to 7, 12-dioxoLCA requires both 7α-dehydrogenation and 12α-dehydrogenation on the same molecule; while we identified 5 strains capable of performing both dehydrogenations on CA, only *H. filiformis* produced the doubly modified bile acid (Fig. 2E).

### Specificity of α-hydroxysteroid dehydrogenase activity.

A comparison between the amounts of secondary bile acids produced by HSD activity at the 3α-, 7α-, and 12α-positions within each strain revealed interesting trends (Fig. 4B). For example, in nearly all isolates, 3α-HSD and 12α-HSD activity exhibited preference toward DCA and/or CDCA over CA (Fig. 4B). For 7α-HSD activity, there was a slight preference toward CDCA over CA for most microbes. However, some isolates, such as *H. filiformis* and Alistipes indistinctus, showed a clear preference for CA over CDCA, while *C. scindens* and *B. ovatus* exhibited a marked preference for CDCA over CA (Fig. 3 and S2; Table S3).

Bacterial isolates also showed some specificity regarding the types of HSD activity they displayed. That is, of the 42 strains that possessed HSDs, 14 showed only one type of HSD activity (3 species were able to act at C-3, 10 species at C-7, and 1 species at C-12), 21 displayed two types of HSD activity, and only 5 isolates possessed all three types of HSD activity (Fig. 3 and S1).

### 7α-Dehydroxylation activity.

Bile acid 7α-dehydroxylation (e.g., transformation of CA into DCA, and CDCA into LCA) (Fig. 2F) is a multistep pathway performed by the bile acid inducible (*bai*) operon, which encodes seven enzymes and a transporter (11, 54, 55). This pathway is reported to be highly conserved and has primarily been observed in *Clostridia* species (36, 47, 56), but our results suggest it to be more widespread (Fig. 3 and S1).

In our screen, 7α-dehydroxylation of CA to DCA was more prevalent than 7α-dehydroxylation of CDCA to LCA (Fig. 3). We found 5 strains capable of 7α-dehydroxylation: Bacteroides vulgatus, Bifidobacterium adolescentis, and Roseburia intestinalis dehydroxylated CA, *C. scindens* dehydroxylated CDCA, and *C. hylemonae* dehydroxylated both CA and CDCA.

Three previously reported 7α-dehydroxylating strains are *C. hylemonae* DSM 15053, *C. scindens* ATCC 35704, and *C. leptum* ATCC 29065. *C. leptum* ATCC 29065 and *C. scindens* ATCC 35704 have been shown to dehydroxylate both CA and CDCA, while *C. hylemonae* DSM 15053 has been shown to only dehydroxylate CA (27, 42, 57, 58). We were unable to reproduce 7α-dehydroxylation of both CA and CDCA in *C. leptum* ATCC 29065 but observed that *C. hylemonae* DSM 15053 dehydroxylated CA to DCA as previously reported (27, 58). Our *C. scindens* isolate showed inconsistent low-level production of LCA from CDCA and DCA from CA (Fig. 3 and Table S3).

Of all five strains we found to perform 7α-dehydroxylation, only *C. hylemonae* possessed the canonical *bai* operon, encoding BaiA2, BaiB, BaiCD, BaiE, BaiF, BaiG, and BaiH but not BaiI (see Materials and Methods). Interestingly, our lab isolate of *C. scindens*, which displayed only low levels of the expected 7α-dehydroxylation activity, also possessed the canonical *bai* operon without *baiI*. However, when comparing our *C. scindens* isolate to the previously sequenced *C. scindens* VPI 12708, we identified one mismatch in the protein sequences for BaiB (amino acid substitution H214L) and BaiH (amino acid substitution V526A) and two mismatches in BaiE (amino acid substitutions T74S and A95E). It is possible that these alterations were enough to decrease its 7α-dehydroxylation activity, but further analyses will need to be performed to support this hypothesis. Finally, *R. intestinalis*, B. vulgatus, and *B. adolescentis* were all predicted to possess only four or five genes of the canonical eight-gene operon. This suggests that divergent or nonhomologous genes may encode the 7α-dehydroxylation activity in the newly identified strains.

### Other bile acid transformations: hydroxyl group conversions at C-6 and C-7.

Consistent with previous reports, we observed that both *C. aerofaciens* and Ruminococcus gnavus produced small amounts of UDCA from CDCA (Fig. 3 and S2) (28, 29). The transformation of CDCA to UDCA requires the combined action of 7α- and 7β-HSDs to epimerize the C-7 hydroxyl group on the steroid core (Fig. 2A and G) (11). This bile acid transformation is of particular interest, because UDCA is used therapeutically to treat gastrointestinal tract diseases (29, 59).

Another bile acid transformation of interest is conversion of DCA to HDCA (Fig. 2G) (60, 61). Collinsella stercoris produced 1.55 μM HDCA from DCA at 48 h (Fig. 3 and S2). While the enzymes responsible for this transformation are not known, it is almost certainly a multistep mechanism involving α-dehydroxylation of the C-12 hydroxyl group followed by a hydroxylation on C-6.

To the best of our knowledge, production of β-muricholic acid (β-MCA) by human gut isolates has not been previously reported. Here, we found that *R. gnavus* can transform CA to β-MCA (Fig. 2G, 3, and S2). This transformation requires α-dehydroxylation at C-12, epimerization of the C-7 hydroxyl group of CA, and addition of a hydroxyl group at the C-6 position. Interestingly, bacterial 12α-dehydroxylation of bile acids appears to be a rare transformation (62, 63).

### Amino acid conjugation of bile acids by gut bacteria.

**FIG 5:**
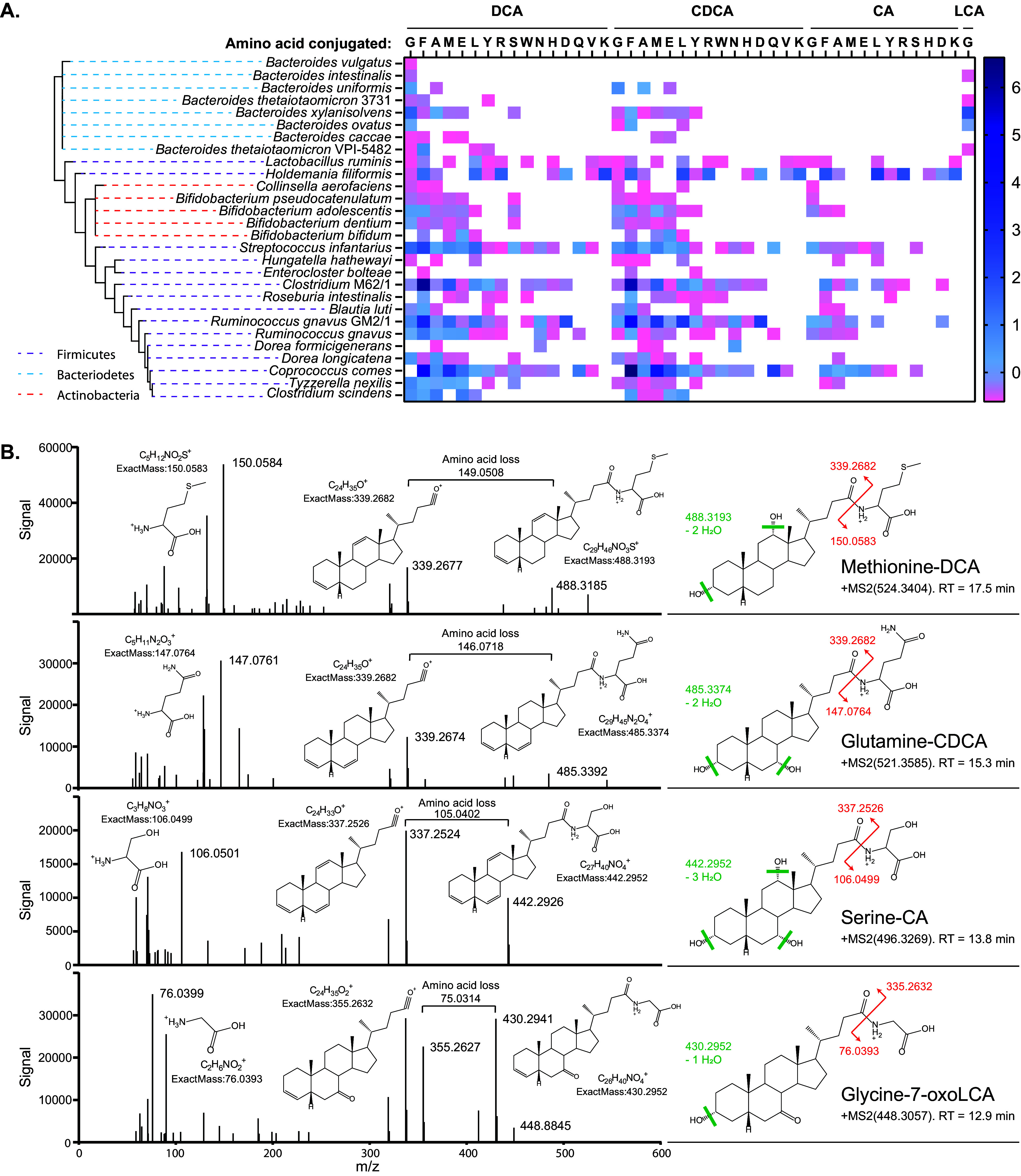
Production of amino acid-conjugated bile acids across bacterial strains. (A) The heat map shows the bile acid-to-amino acid conjugations carried out by each isolate at 24 h. The data are presented as raw signal intensities normalized by Z-score, which is denoted by the color scale. Bacteria were provided with 100 μM cholic acid (CA), chenodeoxycholic acid (CDCA), or deoxycholic acid (DCA). Phyla information is indicated by the color-coded dashed lines in the phylogenetic tree on the left. The heat map for conjugated bile acid production at 48 h is shown in Fig. S3. (B) MS/MS spectra of selected novel microbially conjugated bile acids. Parent ion structure, mass, and retention time are listed along with the structure and exact masses for three identifying fragments: the major sterol fragment, the fragment resulting from amino acid loss, and the amino acid fragment.

Our *in vitro* screen revealed that 25 strains, representing 24 species, were capable of conjugating DCA, CDCA, or CA to glycine *in vitro* (Fig. 5A and Table S4). Twenty-three strains produced up to 1.02 μM (averaging ∼0.236 μM at 48 h) of glycodeoxycholic acid (GDCA) from DCA, 16 strains produced up to 0.70 μM (averaging ∼0.145 μM at 48 h) of glycochenodeoxycholic acid (GCDCA) from CDCA, and 7 strains produced up to 0.098 μM (averaging 0.036 μM at 48 h) of glycocholic acid (GCA) from CA (Fig. 5A and Table S4). To our knowledge, this is the first report of bacterially mediated reconjugation of bile acids to glycine.

10.1128/mSystems.00805-21.8 TABLE S4 Amino acid conjugation data. Download Table S4, XLSX file, 0.042 MB. Copyright © 2021 Lucas et al. 2021 Lucas et al. https://creativecommons.org/licenses/by/4.0/ This content is distributed under the terms of the Creative Commons Attribution 4.0 International license.

10.1128/mSystems.00805-21.3 FIG S3 Production of amino acid-conjugated bile acids across bacterial strains. The heat map shows the bile acid-to-amino acid conjugations carried out by each isolate at 48 h. The data are presented as raw signal intensity normalized by Z-score, which is denoted by the color scale. Bacteria were provided with 100 μM cholic acid (CA), chenodeoxycholic acid (CDCA), or deoxycholic acid (DCA). Phyla information is indicated by the color-coded dashed lines in the phylogenetic tree on the left. Download FIG S3, PDF file, 0.2 MB. Copyright © 2021 Lucas et al. 2021 Lucas et al. https://creativecommons.org/licenses/by/4.0/ This content is distributed under the terms of the Creative Commons Attribution 4.0 International license.

In addition to the microbial conjugation of CDCA, DCA, and CA to glycine, we also observed glycine conjugation to the most prominent secondary bile acid produced in our screen, 7-oxolithocholic acid (7-oxoLCA) (Fig. 5, S3, and Table S4). All strains that could perform this conjugation were members of the *Bacteroidaceae* and produced detectable amounts of glycol-7-oxolithocholic (G-7-oxoLCA) acid when provided with CDCA as a substrate.

A recent study reported that the mouse microbiome is capable of conjugating CA to the amino acids phenylalanine, tyrosine, and leucine and showed that Enterocloster (formerly *Clostridium*) bolteae can carry out CA conjugations to phenylalanine and tyrosine *in vitro* (15). Guided by these data, we searched our data for the presence of bile acids conjugated to amino acids. We found that 28 isolates, representing 27 species, were capable of conjugating CDCA, DCA, or CA to one or more of the following amino acids: glutamate, glutamine, aspartate, asparagine, methionine, histidine, lysine, serine, tryptophan, valine, alanine, and arginine; they were also able to conjugate to the previously described phenylalanine, leucine/isoleucine, or tyrosine (Fig. 5 and S3). In total, we identified 44 novel amino acid-bile acid conjugates. The amino acids most frequently conjugated to bile acids were glycine and phenylalanine (Fig. 5 and S3). CDCA and DCA were conjugated to amino acids much more frequently than CA. Of these 28 isolates, 25 overlapped with strains that possessed the ability to conjugate bile acids to glycine (Fig. 5, S3, and Table S4). To corroborate the identification of conjugated bile acids, we used tandem mass spectrometry (MS/MS) to generate high-resolution spectra for each conjugated bile acid of interest (Fig. 5B); these spectra displayed the expected fragmentation patterns (Table S5) (15).

10.1128/mSystems.00805-21.9 TABLE S5 Retention time data and MS/MS fragmentation data for conjugated bile acids. Download Table S5, XLSX file, 0.1 MB. Copyright © 2021 Lucas et al. 2021 Lucas et al. https://creativecommons.org/licenses/by/4.0/ This content is distributed under the terms of the Creative Commons Attribution 4.0 International license.

Interestingly, in some bacterial strains, we noticed double chromatographic peaks corresponding to the exact masses and with the expected characteristic MS/MS spectra for bile acids conjugated to alanine or valine (Fig. S4, Table S5). Enantiomers, which have the same mass, can have different retention times; thus, this observation suggests that some bacterial strains are conjugating both d- and l-alanine or valine to CDCA, DCA, or CA (Fig. S4 and Table S5).

10.1128/mSystems.00805-21.4 FIG S4 Extracted MAVEN files showing double peaks for amino acid conjugated bile acids. Raw files were exported to show peaks for suspected l- and d-enantiomers of alanine and valine conjugated to bile acids. Download FIG S4, PDF file, 1 MB. Copyright © 2021 Lucas et al. 2021 Lucas et al. https://creativecommons.org/licenses/by/4.0/ This content is distributed under the terms of the Creative Commons Attribution 4.0 International license.

Our results indicate that the ability to conjugate amino acids to primary and secondary bile acids is widespread among gut microbes (Fig. 5 and S3; Table S4). Bile acid conjugation to taurine was not observed in our screen, either because organisms lack the necessary enzymes or because the growth medium did not supply sufficient amounts of the compound. Further investigation is required to determine the possibility of microbial taurine conjugation to bile acids. The enzymes responsible for amino acid conjugation are currently unknown, and while it is plausible that microbial BSH may participate in glycine conjugation (64), it is unclear what enzymes would be responsible for bile acid conjugation to all other amino acids. Interestingly, we saw most conjugation ability in the *Actinobacteria*, *Firmicutes*, and *Bacteroidetes*, which are the same three phyla reported to possess glycine reconjugation activity and BSH activity. In addition, most species fall into three families: *Bifidobacteriaceae*, *Lachnospiraceae*, and *Bacteroidaceae* (Fig. 5 and S1) (5). The physiological effects of amino acid-conjugated bile acid—other than those conjugated to glycine or taurine—are unknown, although it was recently reported that cholic acid conjugated to phenylalanine, leucine, and tyrosine can act as FXR agonists and are enriched in patients with inflammatory bowel disease and cystic fibrosis (15). In addition, it is unknown whether bile acid conjugation to amino acids other than glycine or taurine enhances their intestinal absorption and enterohepatic circulation. Further studies are required to investigate these important questions.

### Comparison of bioinformatically predicted and observed HSD activity.

A previous study by Kisiela et al. included a bioinformatic search for 3α-, 7α-, and 12α-HSD homologs in all sequenced bacteria and archaea (at the time of analysis) and identified putative HSDs across numerous genera (26). The study, which evaluated 69 of our 72 bacterial strains, used known protein sequences to identify HSD homologues. They predicted 22 of the 40 bacterial species that exhibited HSD activity in this *in vitro* study. We were able to confirm all predicted HSD activities (3α-, 7α-, and 12α-HSD) in all species except two (*C. hylemonae* and *C. scindens*). We confirmed 7 of 8 species predicted to possess 3α-HSD activity, 6 of which were an exact strain match, 10 of 10 species predicted to possess 7α-HSD activity, 8 of which were an exact strain match, and 9 of 10 species predicted to possess 12α-HSD activity, 9 of which were an exact strain match (Fig. S1 and Table S6). Although *C. hylemonae* was predicted to possess both 3α- and 12α-HSD activity, we observed 7α- and 12α-HSD activity in our screen and only trace 3α-HSD activity (i.e., production of 3-oxoCDCA from CDCA of less than 0.1 μM). Interestingly, prior *in vitro* studies on *C. hylemonae* showed only a functional 12α-HSD but not a 3α- or 7α-HSD (27). In addition, *C. scindens* was predicted to possess 3α-, 7α-, and 12α-HSD activity, but in our lab isolate, under the specified experimental conditions, we only observed 3α- and 7α-HSD. Of the 69 sequenced species that overlap between our *in vitro* study and the Kisiela et al. bioinformatic analysis (26), we found many species that displayed HSD activity that were not predicted by their analysis (see Table S6). We identified 13 additional species (13 strains) with 3α-HSD activity, 24 additional species (26 strains) with 7α-HSD activity, and 2 additional species (3 strains) with 12α-HSD activity (Fig. S1 and Table S6). This suggests that there are yet to be identified HSDs with low or no homology to currently known enzymes or that some low-level transformations may represent nonspecific enzyme catalysis.

10.1128/mSystems.00805-21.10 TABLE S6 Comparison of observed bile acid transformation in this study and bioinformatic predictions of HSD activity. Download Table S6, XLSX file, 0.1 MB. Copyright © 2021 Lucas et al. 2021 Lucas et al. https://creativecommons.org/licenses/by/4.0/ This content is distributed under the terms of the Creative Commons Attribution 4.0 International license.

A different study by Doden et al. used a bioinformatics approach to identify bacteria with previously unreported 12α-HSD activity (27). They predicted 12α-HSDs to be widespread among the phyla *Firmicutes* and *Actinobacteria*. Our results confirmed the predicted 12α-HSD activity in 11 species identified by their analysis (Fig. S1 and Table S6). Interestingly, their study predicted 12α-HSD activity in 10 species belonging to the phylum *Bacteroidetes* that we analyzed in our *in vitro* screen, but we only observed 12α-HSD activity in two of them. The reason for this discrepancy is unknown, but it is plausible that *Bacteroidetes* species only induce 12α-HSD activity under specific growth conditions, and the specific medium used in this study (see Materials and Methods) may have suppressed the expression of these class of enzymes. It is worth pointing out that the study by Kisiela et al. (26) only identified two *Bacteroidetes* strains with 12-HSD activity, Bacteroides pectinophilus and Bacteroides fragilis (neither of which were assessed in our study), while Doden et al. identified a large number of *Bacteroidetes* strains with this activity, including both aforementioned strains and 12 of the species analyzed in this study (Table S6) (27). The discrepancies between these two studies are likely a reflection of the distinct methodologies used and the specific selection of seed gene sequences, but they also highlight the difficulty in accurately predicting HSD activity from gene sequence homology.

To provide further insight into this issue, we performed a bioinformatic search to predict HSD proteins in our strains based on known sequences (Table S6) (see Materials and Methods). As summarized in Table S6, the predictions of our own bioinformatic analysis showed fair overlap with the Kisiela et al. (26) and Doden et al. (27) studies but still failed to predict the observed *in vitro* bile acid-transforming HSD activity of many species. Overall, these results suggest that nonhomologous genes may be responsible for the observed *in vitro* HSD activity, but further research is required to determine what combination of mutations, deletions, gene duplications, or gene regulation might be responsible for causing discrepancies between observed and predicted HSD activity. In addition, bile acid-transforming activity may be influenced by the gut environment and interspecies interactions *in vivo.*

### Conclusion.

Our results greatly expand upon the lists of organisms known to perform different types of bile acid transformations. The high prevalence of bile acid transformations, with few discernible patterns based on phylogeny, illustrates the widespread distribution of bile acid-transforming capabilities across human gut bacteria. We anticipate that knowledge generated by this study will facilitate engineering of synthetic microbial communities with predictable effects on bile acid composition in *in vivo* systems, which will be vital for assessing the physiological effects of microbially transformed bile acids. A new suite of questions can now be asked about how bacteria act by themselves or in concert with others to transform bile acids and generate a diverse bile acid pool.

## MATERIALS AND METHODS

### Strains and media.

All strains are listed in Table S1 in the supplemental material. All strains were grown on Mega medium, which was filter sterilized and stored in a Coy anaerobic chamber (5% H_2_, 20% CO_2_, and 75% N_2_) at least 24 h prior to use. Mega medium contains (per liter tap distilled water): 100 ml (1 M, pH 7.2) potassium phosphate buffer, 10 g tryptone peptone, 5 g yeast extract, 5 g meat extract, 4 ml (25 mg/100 ml) resazurin, 1.8 g d-glucose, 0.9 g d-maltose, 0.86 g d-cellobiose, 0.46 g d-fructose, 1 g sodium acetate trihydrate, 0.02 g MgSO_4_·7H_2_O, 2.1 g NaHCO_3_, 0.08 g NaCl, 1 ml (0.8 g/100 ml) CaCl_2_, 1 ml (1 mg/ml in 100% ethanol) vitamin K_3_ (menadione), 1 ml (1.2 mg hematin/ml in 0.2 M histidine, pH 8.0) histidine hematin, 2 ml (25% [vol/vol]) Tween 80, 10 ml ATCC MD-VS vitamin mix, 10 ml ATCC MD-TMS trace mineral mix, 1 ml (40 mg/100 ml) FeSO_4_·7H_2_O, and 0.5 g l-cysteine HCl. For cultures of Akkermansia muciniphila, the medium was amended with 0.5 mg/ml mucin. This specific medium was designed to allow growth of all species in this study. Clostridium scindens was isolated from a quercetin-degrading anaerobic enrichment inoculated with a human fecal sample (65). The *C. scindens* genome was sequenced by the Microbial Genome Sequencing Center (MiGS, Pittsburgh PA). Genome hybrid assembly with Illumina and Oxford Nanopore Technologies (ONT) reads was performed using he Unicycler v.0.4.8 pipeline; 1.083 Gbp Illumina reads and 1.004 Gbp ONT reads were assembled into a single contig of 3,941,835 bp (530× coverage) with 47.61% GC content. Assembly annotation was performed using Prokka v.1.14.5. The genome sequence data of the *C. scindens* strain isolated in this study can be accessed with the NCBI reference sequence NZ_CP080442.1.

### Sample handling and growth conditions.

Strains were grown at 37°C in an anaerobic chamber with an atmosphere of 75% N_2_, 20% CO_2_, and 5% H_2._ Starting from freezer stocks, strains were first grown overnight to a high density (optical density at 600 nm [OD_600_] range of 0.349 to 1.9, measured directly in the tube) in Hungate tubes containing Mega medium. These cultures were then used to inoculate (1:15 dilution) 3 ml of Mega medium in 5-ml polypropylene tubes, amended with bile acids. There were 5 sets of conditions for the quantitative screen of bile acid-transforming activity. Under the first three conditions, medium contained one of each of the three bile acids, cholic acid (CA), chenodeoxycholic acid (CDCA), or deoxycholic acid (DCA), at 100 μM each. Under the fourth condition, all three bile acids were combined, totaling 300 μM. Under the fifth condition (control), no bile acids were provided. In addition, we tested for spontaneous bile acid degradation or transformation in uninoculated controls containing bile acids. At 24 and 48 h after inoculation, 1 ml of culture was collected and spun down at room temperature for 10 min at 10,000 × *g*, and the supernatant was transferred to a fresh tube. The supernatant was diluted to 1:100 using ultrahigh-pressure liquid chromatography (uHPLC)-grade H_2_O, and 100 μl was transferred to an HPLC vial for analysis. In addition to the qualitative screen, we also carried out an initial qualitative screen in which strains were grown in 96-well plates (200-μl cultures) for 24 h with bile acids mixed totaling 300 μM. In combination, 5 independent measurements of bile acid-transforming activity were carried out for each bacterial strain in this study: four quantitative measurements and an initial qualitative assessment.

### uHPLC-MS/MS measurements.

Samples were analyzed using an ultrahigh-pressure liquid chromatography-tandem mass spectrometry (uHPLC-MS/MS) system consisting of a Thermo Scientific Vanquish uHPLC system coupled to a heated electrospray ionization (HESI; using negative polarity) and hybrid quadrupole high-resolution mass spectrometer (Q Exactive Orbitrap; Thermo Scientific). Settings for the ion source were as follows: auxiliary gas flow rate of 10, sheath gas flow rate of 30, sweep gas flowrate of 1, 2.5-kV spray voltage, 320°C capillary temperature, 300°C heater temperature, and S-lens radiofrequency (RF) level of 50%. Nitrogen was used as nebulizing gas by the ion trap source. Liquid chromatography (LC) separation was achieved using a Waters Acquity UPLC BEH C_18_ column with 1.7-μm particle size, 2.1 by 100 mm in length. Solvent A was water with 10 mM ammonium acetate adjusted to pH 6.0 with acetic acid. Solvent B was 100% methanol. The total run time was 31.5 min with the following gradient: a 0- to 24-min gradient from 30% solvent B (initial condition) to 100% solvent B; hold 5 min at 100% solvent B; drop to 30% solvent B for 2.5-min reequilibration to initial condition. The flow rate was 200 μl/min throughout. Other LC parameters were as follows: autosampler temperature, 4°C; injection volume, 10 μl; column temperature, 50°C. The MS method performed a full MS1 full scan (290 to 1,000 *m/z*) together with a series of parallel reaction monitoring (PRM) scans. These MS2 scans (all-ion fragmentation) were centered at *m/z* values of 370, 408, 446, 484, and 522; each using an isolation width of 40.0 *m/z*. Fragmentations were performed at 60 normalized collision energy (NCE). All scans used a resolution value of 70,000, an automatic gain control (ACG) target value of 1E6, and a maximum injection time (IT) of 40 ms. Experimental MS data were converted to the mzXML format and used for bile acid identification. Bile acid peaks were identified using MAVEN (metabolomics analysis and visualization engine) (66, 67). For tandem mass spectrometry of conjugated bile acids, MS2 scans (selected ion fragmentation) were performed at 20, 30, and 40 NCE (normalized collision energy). All scans used a resolution value of 17,500, an automatic gain control (ACG) target value of 1E6, and a maximum injection time (IT) of 40 ms.

### Determination of bile acid concentrations.

Bile acid quantitation was achieved using standard concentrations of each bile acid ranging from 0.01 μg/ml (or 0.019 μM to 0.027 μM depending on the bile acid) to 1 μg/ml (or 1.94 μM to 2.65 μM depending on the bile acid) to generate five-point standard curves. The detection limit was below 0.05 μM for all bile acids. The threshold for reported core bile acid transformations was 0.1 μM. Standards were purchased from Avanti Polar Lipids and dissolved and stored in methanol at −80°C. See Table S2 for bile acid standard names and structural features. For bile acids conjugated to amino acids, compounds were identified by their exact mass (mass error of less than 2 ppm) and predicted retention times but could not be quantified, since standards are not commercially available (Table S5). The only exceptions were bile acids conjugated to glycine (i.e., GDCA and CDCA), for which standards were available.

### BLASTP search for the *bai* operon

Bacterial genomes were downloaded from NCBI ftp site using GenBank assemblies (GCA) or RefSeq assemblies (GCF). Eight amino acid sequences from Clostridium scindens VPI 12708 were downloaded from UniProt and used as reference sequences: BaiB (P19409), BaiCD (P19410), BaiE (P19412), BaiA2 (P19413), BaiF (P19413), BaiG (P32369), BaiH (P32370), and BaiI (P32371). Each of these eight Bai proteins were searched against all protein coding genes (CDS) from the downloaded bacterial genomes, with an E value threshold of 1E−10. Gene coordinates (start position, end position, strand) in the genome were obtained from the GFF file on the NCBI ftp site. A *bai* operon was considered highly homologous if gene queries yielded eight *bai* operon genes within a close genome region (<100,000 bp).

### Hidden Markov model search for the HSD gene.

Bacterial genomes were downloaded from the NCBI ftp site using GenBank assemblies (GCA) or RefSeq assemblies (GCF). To annotate 3α-, 7α-, 12α-HSD genes, hidden Markov model (HMM) searches were performed using custom HMM profiles against all protein coding genes (CDS) from the downloaded bacterial genomes. To generate 3α-, 7α-, 12α-HSD HMM profiles, reference sequences were downloaded from UniProt, and only reviewed (Swiss-Prot) sequences or sequences validated by experiments in the literature were used: for 3α-HSD, reference sequences included Q59718 (Pseudomonas sp. B-0831), P80702 (Pseudomonas testosteroni), A7B3K3 (Ruminococcus gnavus strain ATCC 29149/VPI C7-9), C8WMP0 (Eggerthella lenta), P19337 (Clostridium scindens strain JCM 10418/VPI 12708), and P07914 (Clostridium scindens strain JCM 10418/VPI 12708). For 7α-HSD, reference sequences included P0AET8 (Escherichia coli strain K-12), Q8YIN7 (Brucella melitensis biotype 1 strain 16M/ATCC 23456/NCTC 10094), Q5LA59 (Bacteroides fragilis strain ATCC 25285/DSM 2151/JCM 11019/NCTC 9343), G9FRD7 (Clostridium absonum), and P50200 (Clostridium sordellii). For 12α-HSD, reference sequences validated by Doden et al. (27) were used, including C0BWQ2 (Clostridium hylemonae DSM 15053), P21215 (*Clostridium* sp. strain ATCC 29733/VPI C48-50), R7AM69 (*Eggerthella* sp. CAG:298), C8WLK7 (Eubacterium lentum strain ATCC 25559/DSM 2243/JCM 9979/NCTC 11813/VPI 0255), B0NG52 (Clostridium scindens strain ATCC 35704/DSM 5676/VPI 13733/19), and B6FYX7 (Clostridium hiranonis strain DSM 13275/JCM 10541/KCTC 15199/TO-931). Reference sequences from each HSD were aligned using MUSCLE (68) version 3.8.31, and the HMM profile was constructed using hmmbuild3 (69). All CDS in bacterial genomes were searched using HMM search version 3.2.1. The identified cutoff in each profile HMM was determined by a minimal bit score to maximize the F measure. In each 3α-, 7α-, and 12α-HSD profile HMM, the positive group was defined as its reference sequences, and the negative group was defined as a combination of the other two references. For example, the positive group for the 3α-HSD profile HMM was six 3α-HSD reference sequences, and the negative group for the 3α-HSD profile HMM was the combined 7α- and 12α-HSD reference sequences. The F measure score was defined as follows: F = 2/(recall^−1^ + precision^−1^), where “recall” is the true positive/(true positive + false negative) and “precision” is the true positive/(true positive + false positive). By this procedure, the cutoff for 3α-HSD was 146.1, the cutoff for 7α-HSD was 220.6, and the cutoff for 3α-HSD was 120.2.

All identified HSD genes together with HMM reference sequences were aligned with MUSCLE (68) version 3.8.31. Conserved motif sequences were visualized by WebLogo3 (70).

### Phylogenetic tree construction.

We used publicly available full genomes and constructed the tree using the Genome Clustering tool using the Joint Genome Institute’s IMG/MER, which is based on hierarchical clustering of the taxonomy of selected genera (Fig. S1; Table S1) (71, 72).

### Data availability.

The HMM search file is publicly available at https://github.com/qijunz/Lucas_BA_paper. *C. scindens* genome sequence data have been deposited in NCBI under reference sequence NZ_CP080442.1.
