# Supplementary material for: Dominant Bacterial Phyla from the Human Gut Show Widespread Ability To Transform and Conjugate Bile Acids
Source: mSystems. 2021 Aug 31;6(4):10.1128/msystems.00805-21. doi: 10.1128/msystems.00805-21 (PMC12338150; doi:10.1128/msystems.00805-21)
Supplement: TABLE S2 [file msystems.00805-21-st002.docx]

| **Supplementary Table 2. Bile acid names and structure description for standards used for LC-MS/MS analysis.** | | | | | | | | | | | |
| --- | --- | --- | --- | --- | --- | --- | --- | --- | --- | --- | --- |
| **Bile Acid Common Name** | **Abbreviation** | **Mol. Formula** | **C1** | **C2** | **C3** | **C6** | **C7** | **C12** | **DB** | **R** | **Rt** |
| Glycocholic acid | GCA | C26H43NO6 | α,β-H | α,β-H | α-OH | α,β-H | α-OH | α-OH | N/A | G | 14.05 |
| Taurocholic acid | TCA | C26H45NO7S | α,β-H | α,β-H | α-OH | α,β-H | α-OH | α-OH | N/A | T | 13.9 |
| Cholic acid | CA | C24H40O5 | α,β-H | α,β-H | α-OH | α,β-H | α-OH | α-OH | N/A | H | 15.04 |
| Glycochenodeoxycholic acid | GCDCA | C26H43NO5 | α,β-H | α,β-H | α-OH | α,β-H | α-OH | α,β-H | N/A | G | 15.94 |
| Taurochenodeoxycholic acid | TCDCA | C26H45NO6S | α,β-H | α,β-H | α-OH | α,β-H | α-OH | α,β-H | N/A | T | 15.73 |
| Chenodeoxycholic acid | CDCA | C24H40O4 | α,β-H | α,β-H | α-OH | α,β-H | α-OH | α,β-H | N/A | H | 17.1 |
| Glycoursodeoxycholic acid | GUDCA | C26H43NO5 | α,β-H | α,β-H | α-OH | α,β-H | β-OH | α,β-H | N/A | G | 12.51 |
| Tauroursodeoxycholic acid | TUDCA | C26H45NO6S | α,β-H | α,β-H | α-OH | α,β-H | β-OH | α,β-H | N/A | T | 12.45 |
| Ursodeoxycholic acid | UDCA | C24H40O4 | α,β-H | α,β-H | α-OH | α,β-H | β-OH | α,β-H | N/A | H | 13.75 |
| Glycodeoxycholic acid | GDCA | C26H43NO5 | α,β-H | α,β-H | α-OH | α,β-H | α,β-H | α-OH | N/A | G | 16.32 |
| Taurodeoxycholic acid | TDCA | C26H45NO6S | α,β-H | α,β-H | α-OH | α,β-H | α,β-H | α-OH | N/A | T | 16.17 |
| Deoxycholic acid | DCA | C24H40O4 | α,β-H | α,β-H | α-OH | α,β-H | α,β-H | α-OH | N/A | H | 17.46 |
| Glycolithocholic acid | GLCA | C26H43NO4 | α,β-H | α,β-H | α-OH | α,β-H | α,β-H | α,β-H | N/A | G | 17.75 |
| Taurolithocholic acid | TLCA | C26H45NO5S | α,β-H | α,β-H | α-OH | α,β-H | α,β-H | α,β-H | N/A | T | 17.61 |
| Lithocholic acid | LCA | C24H40O3 | α,β-H | α,β-H | α-OH | α,β-H | α,β-H | α,β-H | N/A | H | 19.09 |
| Hyocholic acid/γ-Muricholic acid | HCA/γ-MCA | C24H40O5 | α,β-H | α,β-H | α-OH | α-OH | α-OH | α,β-H | N/A | H | 13.7 |
| Tauro-α-muricholic acid | T-α-MCA | C26H45NO7S | α,β-H | α,β-H | α-OH | β-OH | α-OH | α,β-H | N/A | T | 10.51 |
| α-muricholic acid | α-MCA | C24H40O5 | α,β-H | α,β-H | α-OH | β-OH | α-OH | α,β-H | N/A | H | 11.83 |
| Tauro-β-muricholic acid | T-β-MCA | C26H45NO7S | α,β-H | α,β-H | α-OH | β-OH | β-OH | α,β-H | N/A | T | 10.65 |
| β-muricholic acid | β-MCA | C24H40O5 | α,β-H | α,β-H | α-OH | β-OH | β-OH | α,β-H | N/A | H | 12.03 |
| Tauro-ω-muricholic acid | T-ω-MCA | C26H45NO7S | α,β-H | α,β-H | α-OH | α-OH | β-OH | α,β-H | N/A | T | 12.36 |
| ω-muricholic acid | ω-MCA | C24H40O5 | α,β-H | α,β-H | α-OH | α-OH | β-OH | α,β-H | N/A | H | 11.89 |
| Taurohyodeoxycholic acid | THDCA | C26H45NO6S | α,β-H | α,β-H | α-OH | α-OH | α,β-H | α,β-H | N/A | T | 12.94 |
| Hyodeoxycholic acid | HDCA | C24H40O4 | α,β-H | α,β-H | α-OH | α-OH | α,β-H | α,β-H | N/A | H | 14.34 |
| 3-oxocholic acid | 3-oxoCA | C24H38O5 | α,β-H | α,β-H | =O | α,β-H | α-OH | α-OH | N/A | H | 13.41 |
| 7-oxodeoxycholic acid | 7-oxoDCA | C24H38O5 | α,β-H | α,β-H | α-OH | α,β-H | =O | α-OH | N/A | H | 11.83 |
| 12-oxochenodeoxycholic acid | 12-oxoCDCA | C24H38O5 | α,β-H | α,β-H | α-OH | α,β-H | α-OH | =O | N/A | H | 12.15 |
| 3-oxochenodeoxycholic acid | 3-oxoCDCA | C24H38O4 | α,β-H | α,β-H | =O | α,β-H | α-OH | α,β-H | N/A | H | 15.83 |
| 3-oxodeoxycholic acid | 3-oxoDCA | C24H38O4 | α,β-H | α,β-H | =O | α,β-H | α,β-H | α-OH | N/A | H | 16.13 |
| Isolithocholic acid | isoLCA | C24H40O3 | α,β-H | α,β-H | β-OH | α,β-H | α,β-H | α,β-H | N/A | H | 17.27 |
| 7-oxolithocholic acid | 7-oxoLCA | C24H38O4 | α,β-H | α,β-H | α-OH | α,β-H | =O | α,β-H | N/A | H | 14.13 |
| 12-oxolithocholic acid | 12-oxoLCA | C24H38O4 | α,β-H | α,β-H | α-OH | α,β-H | α,β-H | =O | N/A | H | 14.63 |
| Taurodehydrocholic acid | T-dhCA | C26H39NO7S | α,β-H | α,β-H | =O | α,β-H | =O | =O | N/A | T | 5.98 |
| Glycodehydrocholic acid | G-dhCA | C26H37NO6 | α,β-H | α,β-H | =O | α,β-H | =O | =O | N/A | G | 5.87 |
| Ursocholianic acid | UCA | C24H40O2 | α,β-H | α,β-H | α,β-H | α,β-H | α,β-H | α,β-H | N/A | H | 24.63 |
| Isodeoxycholic acid | isoDCA | C24H40O4 | α,β-H | α,β-H | α,β-H | α,β-H | α-OH | α-OH | N/A | H | 18.49 |
| 6,7-dioxolithocholic acid | 6,7-dioxoLCA | C24H36O5 | α,β-H | α,β-H | α-OH | =O | =O | α,β-H | N/A | H | 14.08 |
| 7,12-dioxolithocholic acid | 7,12-dioxoLCA | C24H36O5 | α,β-H | α,β-H | α-OH | α,β-H | =O | =O | N/A | H | 8.18 |
| Apocholic acid | ACA | C24H38O4 | α,β-H | α,β-H | α-OH | α,β-H | α,β-H | α-OH | 8-14 | H | 16.03 |
| Rt = retention time. R represents side chain abbreviations: G = glycine, T = taurine, H = hydrogen. DB = double bond. | | | | | | | | | | | |
| For numbered carbons on steroid core, see Figure 2. | | | | | | | | | | | |
