# Supplementary material for: Dominant Bacterial Phyla from the Human Gut Show Widespread Ability To Transform and Conjugate Bile Acids
Source: mSystems. 2021 Aug 31;6(4):10.1128/msystems.00805-21. doi: 10.1128/msystems.00805-21 (PMC12338150; doi:10.1128/msystems.00805-21)
Supplement: TABLE S1 [file msystems.00805-21-st001.docx]

| **Supplementary Table 1. Bacterial strain names, ID number, and accession numbers.** | | | | |  |
| --- | --- | --- | --- | --- | --- |
| **Phylum** | **Genus** | **Species** | **ATCC ID** | **DSM #** | **Accession Number** |
| Actinobacteria | *Bifidobacterium* | *adolescentis* | 15703 | N/A | NC_008618.1 |
|  | *Bifidobacterium* | *angulatum* | 27535 | 20098 | NZ_AP012322.1 |
|  | *Bifidobacterium* | *bifidum* | 29521 | 20456 | NZ_AP012323.1 |
|  | *Bifidobacterium* | *dentium* | 27678 | N/A | GCA_000172135.1 |
|  | *Bifidobacterium* | *pseudocatenulatum* | 27919 | 20438 | NZ_AP012330.1 |
|  | *Collinsella* | *aerofaciens^* | 25986 | 3979 | PRJNA18167 |
|  | *Collinsella* | *intestinalis* | N/A | 13280 | GCA_000156175.1 |
|  | *Collinsella* | *stercoris* | N/A | 13279 | GCA_000156215.1 |
| Bacteroidetes | *Alistipes* | *indistinctus* | N/A | 22520 | NZ_ADLD01000000 |
|  | *Bacteroides* | *thetaiotaomicron* 3731^ | N/A | N/A | GCA_001049535.1 |
|  | *Bacteroides* | *thetaiotaomicron* 7330^ | N/A | N/A | GCA_001314975.1 |
|  | *Bacteroides* | *thetaiotaomicron* VPI-5482^ | 29148 | N/A | GCA_000011065.1 |
|  | *Bacteroides* | *caccae* | 43185 | 19024 | PRJNA18163 |
|  | *Bacteroides* | *cellulosilyticus* | N/A | 14838 | GCA_000158035.1 |
|  | *Bacteroides* | *coprophilus* | N/A | 18228 | GCA_000157915.1 |
|  | *Bacteroides* | *dorei* | N/A | 17855 | GCA_000156075.1 |
|  | *Bacteroides* | *finegoldii* | N/A | 17565 | GCA_000156195.1 |
|  | *Bacteroides* | *intestinalis^* | N/A | 17393 | GCA_000172175.1 |
|  | *Bacteroides* | *ovatus* | 8483 | N/A | GCA_001314995.1 |
|  | *Bacteroides* | *plebeius* | N/A | 17135 | GCA_000187895.1 |
|  | *Bacteroides* | *stercoris* | 43183 | N/A | GCA_000154525.1 |
|  | *Bacteroides* | *uniformis* | 8492 | N/A | GCA_000154205.1 |
|  | *Bacteroides* | *vulgatus* | 8482 | N/A | GCA_000012825.1 |
|  | *Bacteroides* | *xylanisolvens* | N/A | 18836 | GCA_000210075.1 |
|  | *Parabacteroides* | *distasonis* | 8503 | 20701 | NC_009615.1 |
|  | *Parabacteroides* | *johnsonii* | N/A | 18315 | GCA_000156495.1 |
|  | *Parabacteroides* | *merdae* | 43184 | 19495 | GCA_000154105.1 |
| Firmicutes | *Anaerococcus* | *hydrogenalis* | 49630 | 7454 | NZ_ABXA01000000 |
|  | *Blautia* | *hansenii* | 27752 | 20583 | GCF_002222595.2 |
|  | *Blautia* | *luti* | N/A | 14534 | GCA_009707925.1 |
|  | *Clostridium* | *asparagiforme* | N/A | 15981 | GCA_000158075.1 |
|  | *Clostridium* | *hylemonae^* | N/A | 15053 | GCA_008281175.1 |
|  | *Clostridium* | *scindens^* | N/A | N/A | NZ_CP080442.1 |
|  | *Clostridium* | *symbiosum* | 14940 | 934 | GCA_000466485.1 |
|  | *Clostridium* | *leptum^* | 29065 | 753 | GCA_000154345.1 |
|  | *Clostridium* | sp. M62_1 | N/A | N/A | GCF_000159055.1 |
|  | *Clostridium* | *sporogenes* | 15579 | N/A | GCA_000155085.1 |
|  | *Coprococcus* | *comes* | 27758 | N/A | GCA_000155875.1 |
|  | *Dorea* | *formicigenerans* | 27755 | 3992 | GCA_000169235.1 |
|  | *Dorea* | *longicatena* | N/A | 13814 | GCA_000154065.1 |
|  | *Enterocloster* | *bolteae^* | BAA-613 | 15670 | GCA_002234575.2 |
|  | *Erysipelatoclostridium* | *ramosum* | 25582 | 1402 | GCA_000154485.1 |
|  | *Eubacterium* | *rectale* | 33656 | N/A | NC_012781.1 |
|  | *Faecalibacterium* | *prausnitzii* M21/2 | N/A | N/A | GCA_000154385.1 |
|  | *Holdemanella* | *biformis* | 27806 | 3989 | GCA_000156655.1 |
|  | *Holdemania* | *filiformis* | 51649 | 12042 | GCA_000157995.1 |
|  | *Hungatella* | *hathewayi* | N/A | 13479 | GCA_000160095.1 |
|  | *Lachnospira* | *eligens* | 27750 | 3376 | GCA_000146185.1 |
|  | *Lactobacillus* | *ruminis* | 27780 | 20403 | GCA_001008755.1 |
|  | *Marvinbryantia* | *formatexigens* | N/A | 14469 | GCA_000173815.1 |
|  | *Megamonas* | *funiformis* | N/A | 19343 | GCA_010669225.1 |
|  | *Mitsuokella* | *multacida* | 27723 | 20544 | GCA_000155955.1 |
|  | *Roseburia* | *intestinalis* | N/A | 14610 | NZ_LR027880.1 |
|  | *Ruminococcus* | *gnavus^* | 29149 | N/A | NZ_CP027002.1 |
|  | *Ruminococcus* | *lactaris* | 29176 | N/A | GCA_000155205.1 |
|  | *Ruminococcus* | *torques* | 27756 | N/A | GCA_000153925.1 |
|  | *Ruminococcus* | sp. GM2/1 | AY305315 | N/A | PRJNA19927 |
|  | *Streptococcus* | *infantarius* | BAA-102 | N/A | GCA_000154985.1 |
|  | *Subdoligranulum* | *variabile* | N/A | 15176 | GCA_000157955.1 |
|  | *Tyzzerella* | *nexilis* | N/A | 1787 | GCF_000156035.2 |
| Fusobacterium | *Fusobacterium* | *varium* | 8501 | 19868 | NZ_LR134390.1 |
| Lentisphaerae | *Victivallis* | *vadensis* | BAA-548 | 14823 | GCA_003096415.1 |
| Proteobacteria | *Desulfovibrio* | *piger* GOR1 | 29098 | 749 | PRJNA30377 |
|  | *Edwardsiella* | *tarda* | 23685 | N/A | GCA_000163955.1 |
|  | *Citrobacter* | *youngae* | 29220 | N/A | GCA_000155975.1 |
|  | *Enterobacter* | *cancerogenus* | 35316 | N/A | GCA_000155995.1 |
|  | *Escherichia* | *coli* K12 MG1655^ | 47076 | 18039 | GCA_009832885.1 |
|  | *Escherichia* | *fergusonii* | 35469 | 13698 | GCA_000026225.1 |
|  | *Proteus* | *penneri* | 35198 | N/A | GCA_000155835.1 |
|  | *Providencia* | *rettgeri* | N/A | 1131 | GCA_000158055.1 |
|  | *Providencia* | *stuartii* | 25827 | N/A | GCA_000154865.1 |
| Verrucomicrobia | *Akkermansia* | *muciniphila* | BAA-835 | 22959 | NC_010655.1 |
| ^Previously known bile acid transformers. | | | | |  |
