# Supplementary material for: Dominant Bacterial Phyla from the Human Gut Show Widespread Ability To Transform and Conjugate Bile Acids
Source: mSystems. 2021 Aug 31;6(4):10.1128/msystems.00805-21. doi: 10.1128/msystems.00805-21 (PMC12338150; doi:10.1128/msystems.00805-21)
Supplement: FIG S1 [file msystems.00805-21-sf001.pdf]

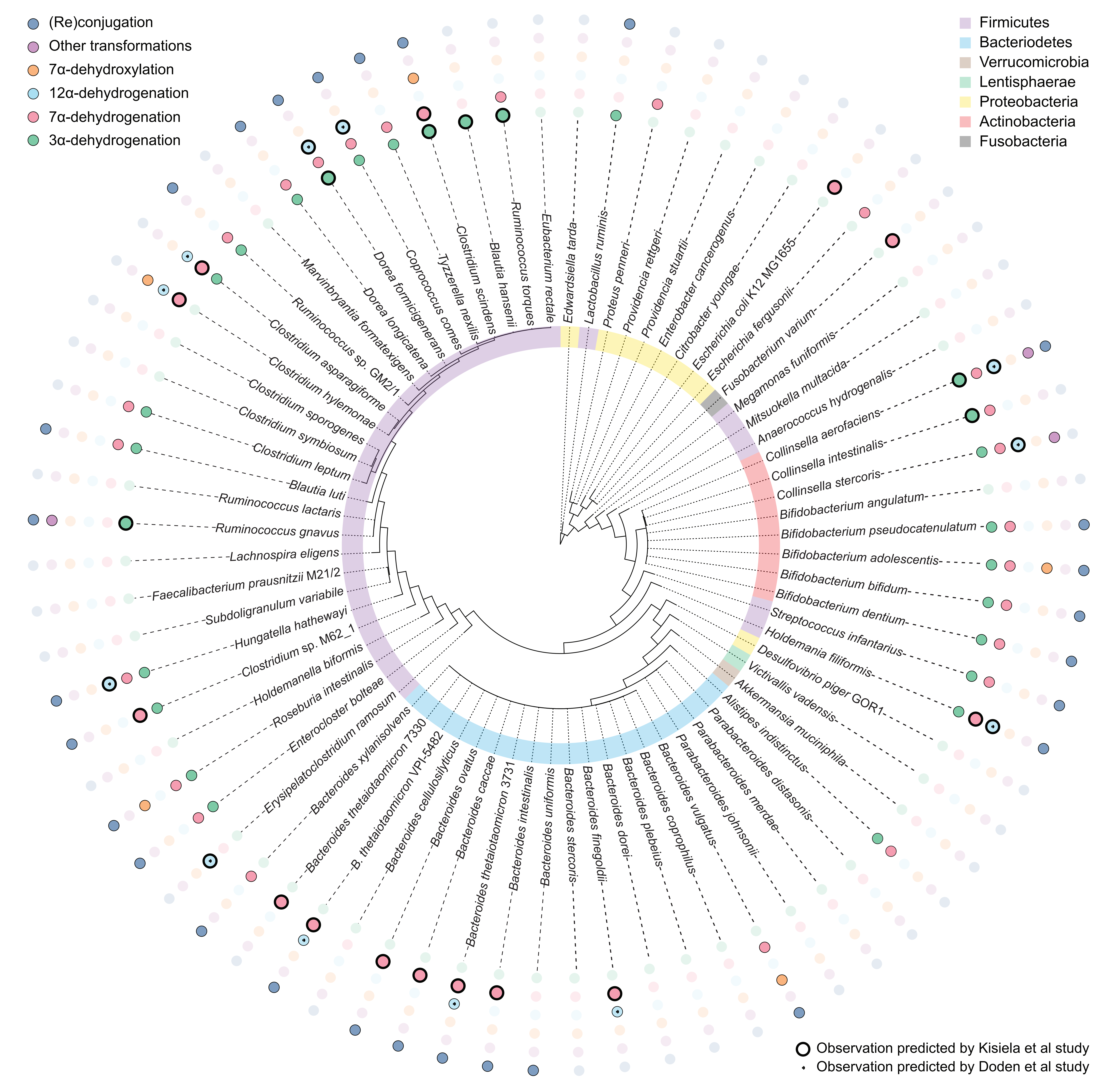

(Re)conjugation
Other transformations
7α-dehydroxylation
12α-dehydrogenation
7α-dehydrogenation
3α-dehydrogenation
Firmicutes
Bacteriodetes
Verrucomicrobia
Lentisphaerae
Proteobacteria
Actinobacteria
Fusobacteria
Eubacterium rectale
Ruminococcus torques
Blautia hansenii
Clostridium scindens
Tyzzerella nexilis
Coprococcus comes
Dorea formicigenerans
Dorea longicatena
Marvinbryantia formatexigens
Ruminococcus sp. GM2/1
Clostridium asparagiforme
Clostridium hylemonae
Clostridium sporogenes
Clostridium symbiosum
Clostridium leptum
Blautia luti
Ruminococcus lactaris
Ruminococcus gnavus
Lachnospira eligens
Faecalibacterium prausnitzii M21/2
Subdoligranulum variabile
Hungatella hathewayi
Clostridium sp. M62_1
Holdemanella biformis
Roseburia intestinalis
Enterocloster bolteae
Erysipelatoclostridium ramosum
Bacteroides xylanisolvens
Bacteroides thetaiotaomicron 7330
B. thetaiotaomicron VPI-5482
Bacteroides cellulosilyticus
Bacteroides ovatus
Bacteroides caccae
Bacteroides thetaiotaomicron 3731
Bacteroides intestinalis
Bacteroides uniformis
Bacteroides stercoris
Bacteroides finegoldii
Bacteroides dorei
Bacteroides plebeius
Bacteroides coprophilus
Bacteroides vulgatus
Parabacteroides johnsonii
Parabacteroides merdae
Parabacteroides distasonis
Alistipes indistinctus
Akkermansia muciniphila
Victivallis vadensis
Desulfovibrio piger GOR1
Holdemania filiformis
Streptococcus infantarius
Bifidobacterium dentium
Bifidobacterium bifidum
Bifidobacterium adolescentis
Bifidobacterium pseudocatenulatum
Bifidobacterium angulatum
Collinsella stercoris
Collinsella intestinalis
Collinsella aerofaciens
Anaerococcus hydrogenalis
Mitsuokella multacida
Megamonas funiformis
Fusobacterium varium
Escherichia fergusonii
Escherichia coli K12 MG1655
Citrobacter youngae
Enterobacter cancerogenus
Providencia stuartii
Providencia rettgeri
Proteus penneri
Lactobacillus ruminis
Edwardsiella tarda
Observation predicted by Kisiela et al study
Observation predicted by Doden et al study
