# Supplementary figures and images for: Dominant Bacterial Phyla from the Human Gut Show Widespread Ability To Transform and Conjugate Bile Acids
Source: mSystems. 2021 Aug 31;6(4):10.1128/msystems.00805-21. doi: 10.1128/msystems.00805-21 (PMC12338150; doi:10.1128/msystems.00805-21)

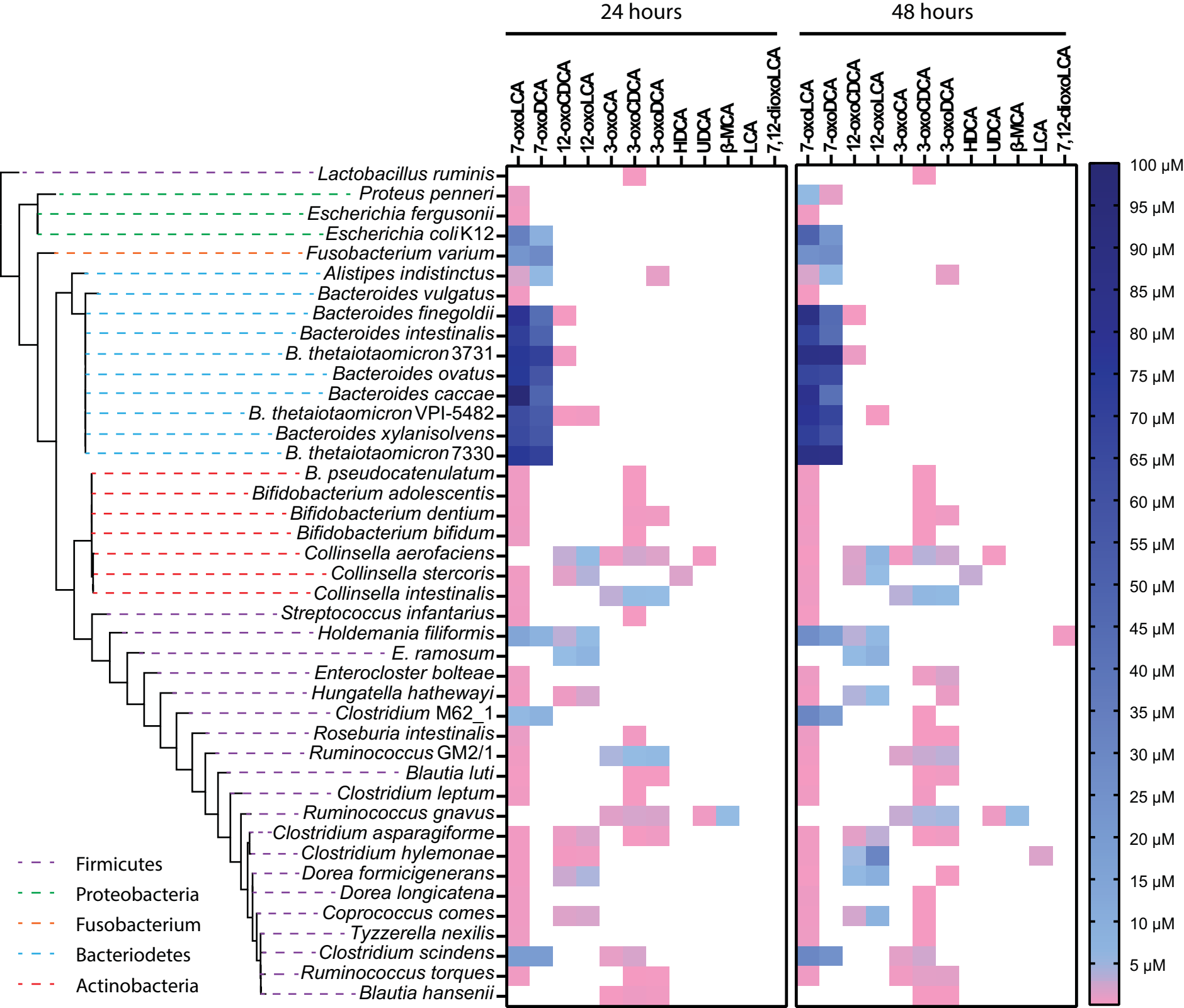

Supplement: FIG S2 [file msystems.00805-21-sf002.pdf]

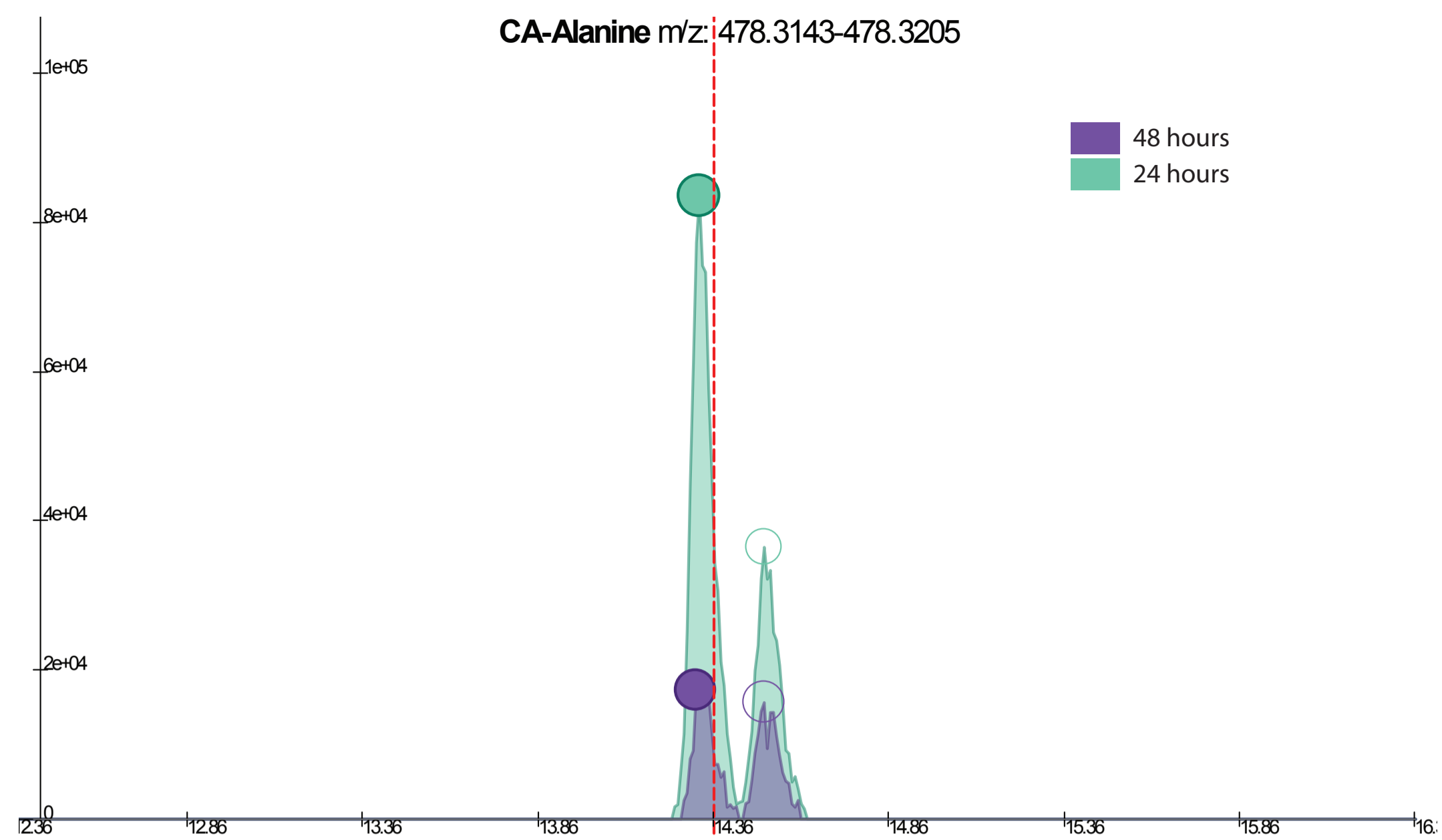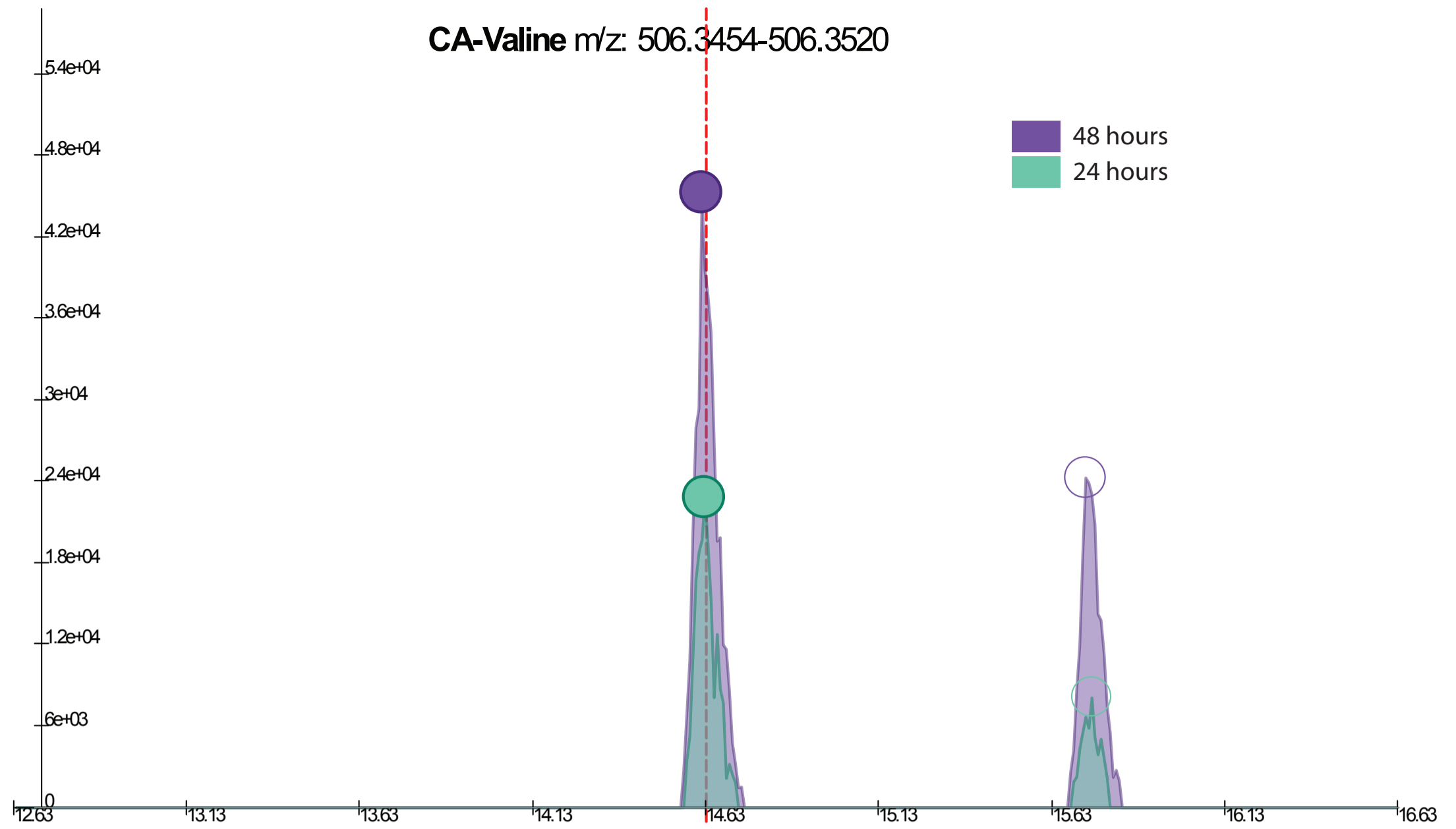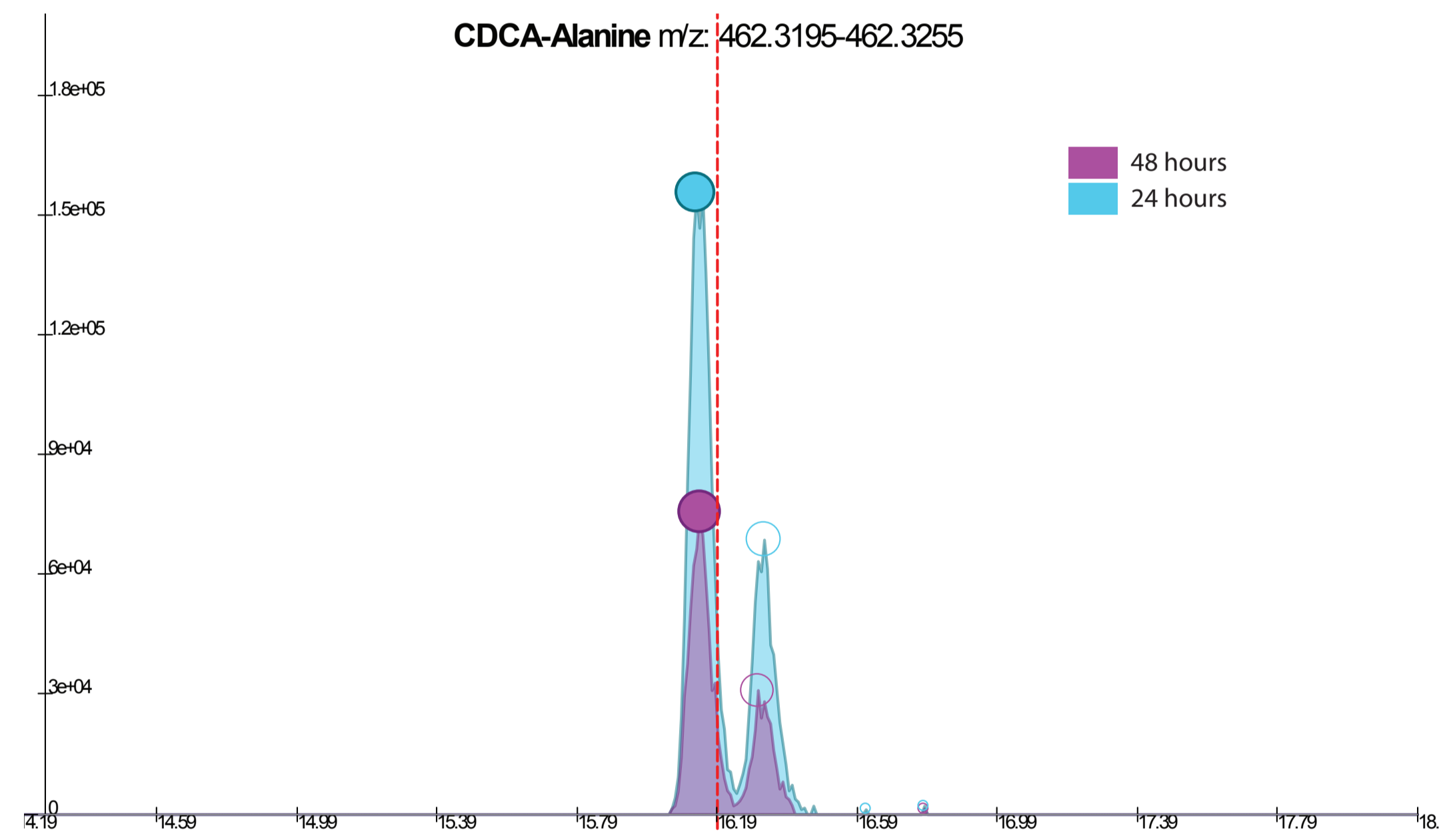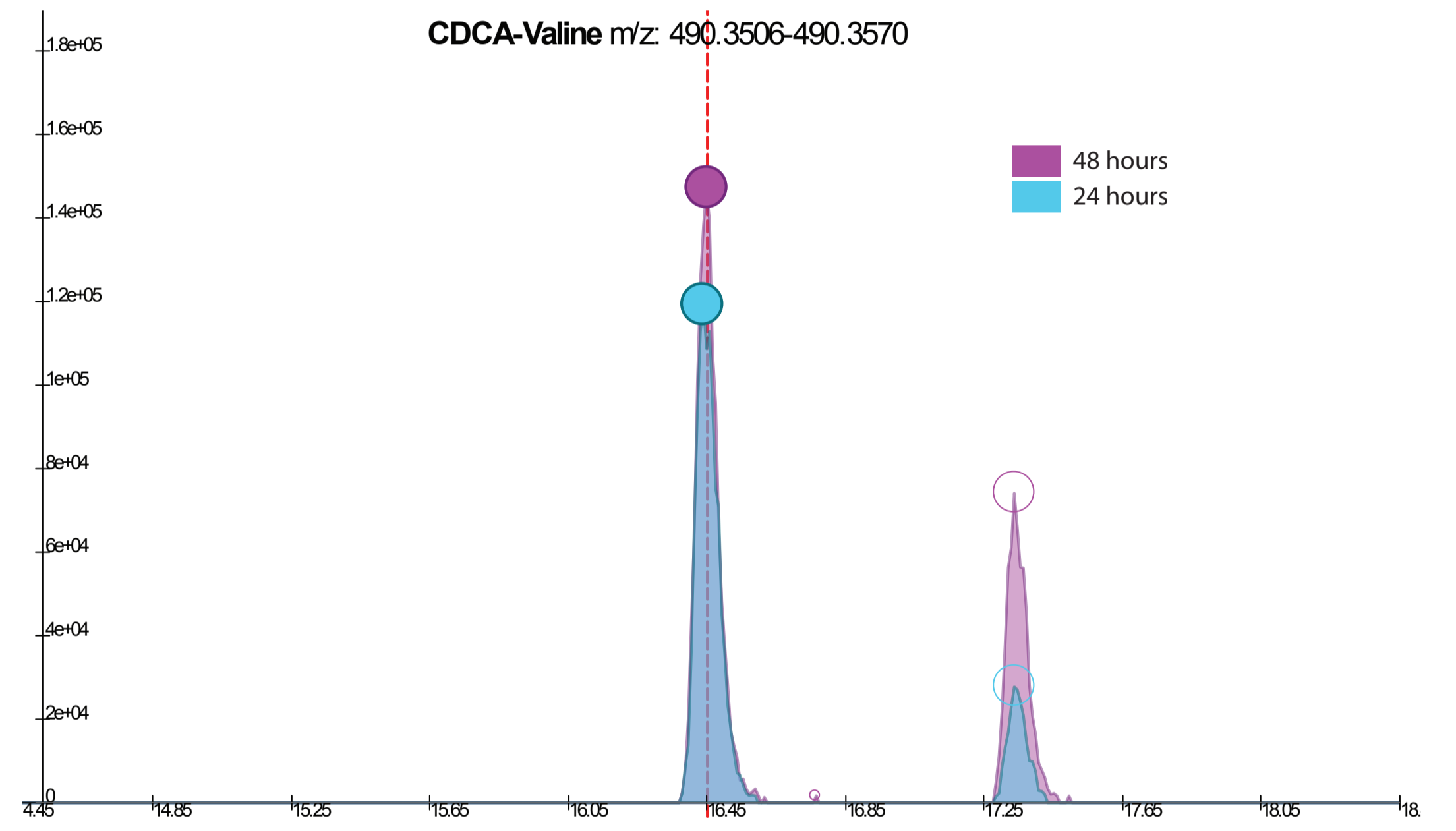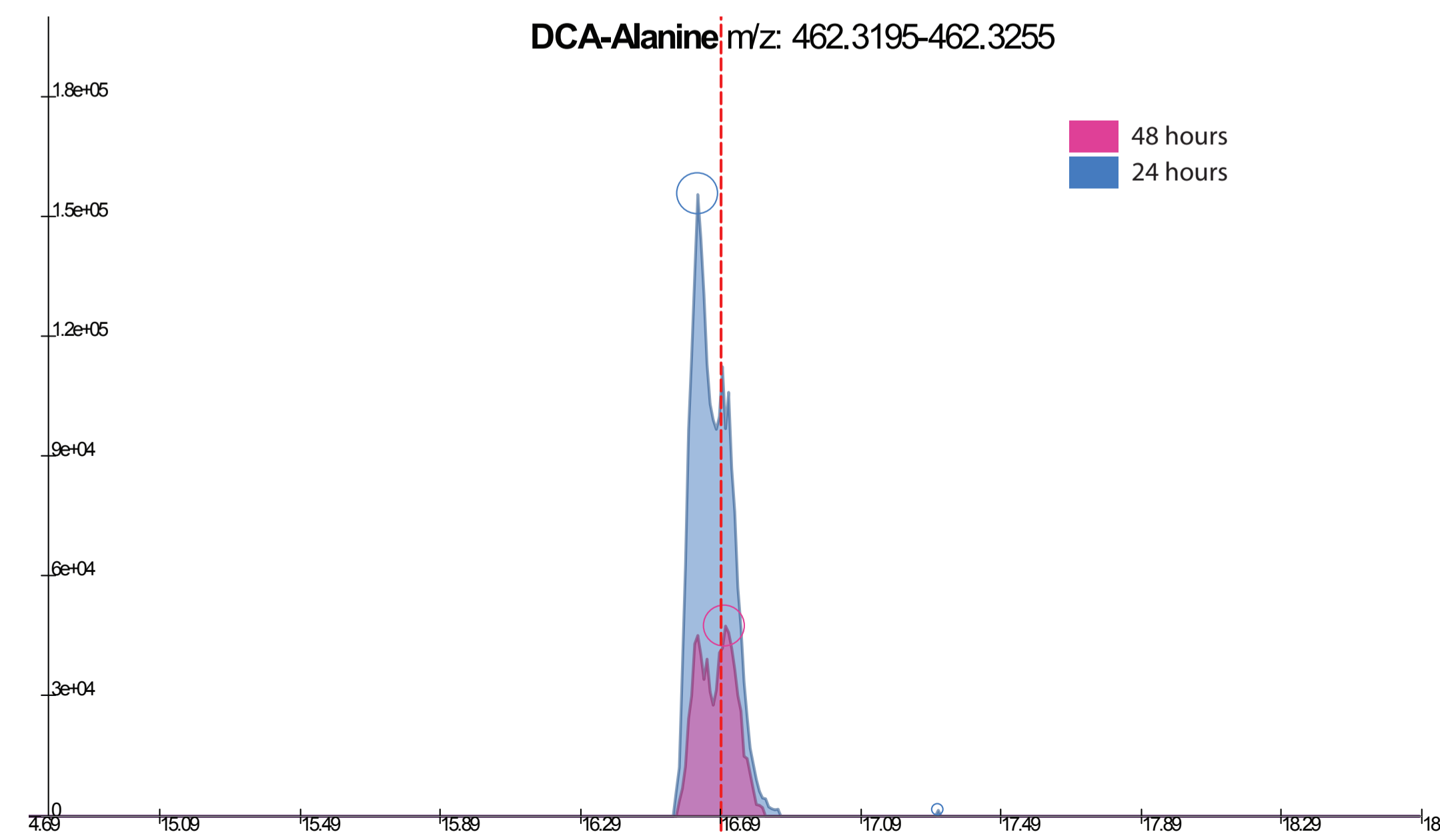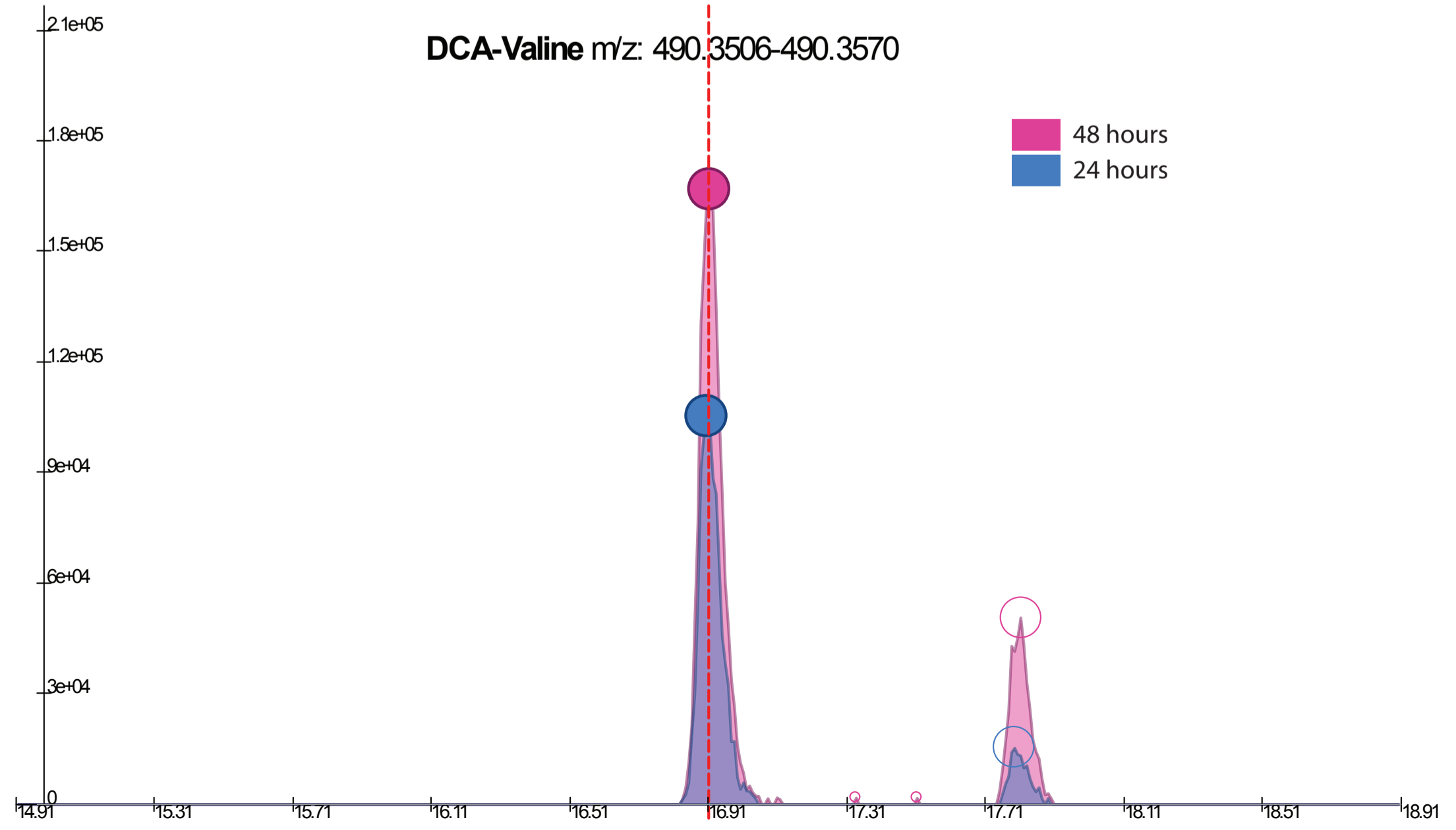

Supplement: FIG S4 [file msystems.00805-21-sf004.pdf]
