# Supplementary material for: Dominant Bacterial Phyla from the Human Gut Show Widespread Ability To Transform and Conjugate Bile Acids
Source: mSystems. 2021 Aug 31;6(4):10.1128/msystems.00805-21. doi: 10.1128/msystems.00805-21 (PMC12338150; doi:10.1128/msystems.00805-21)
Supplement: FIG S3 [file msystems.00805-21-sf003.pdf]

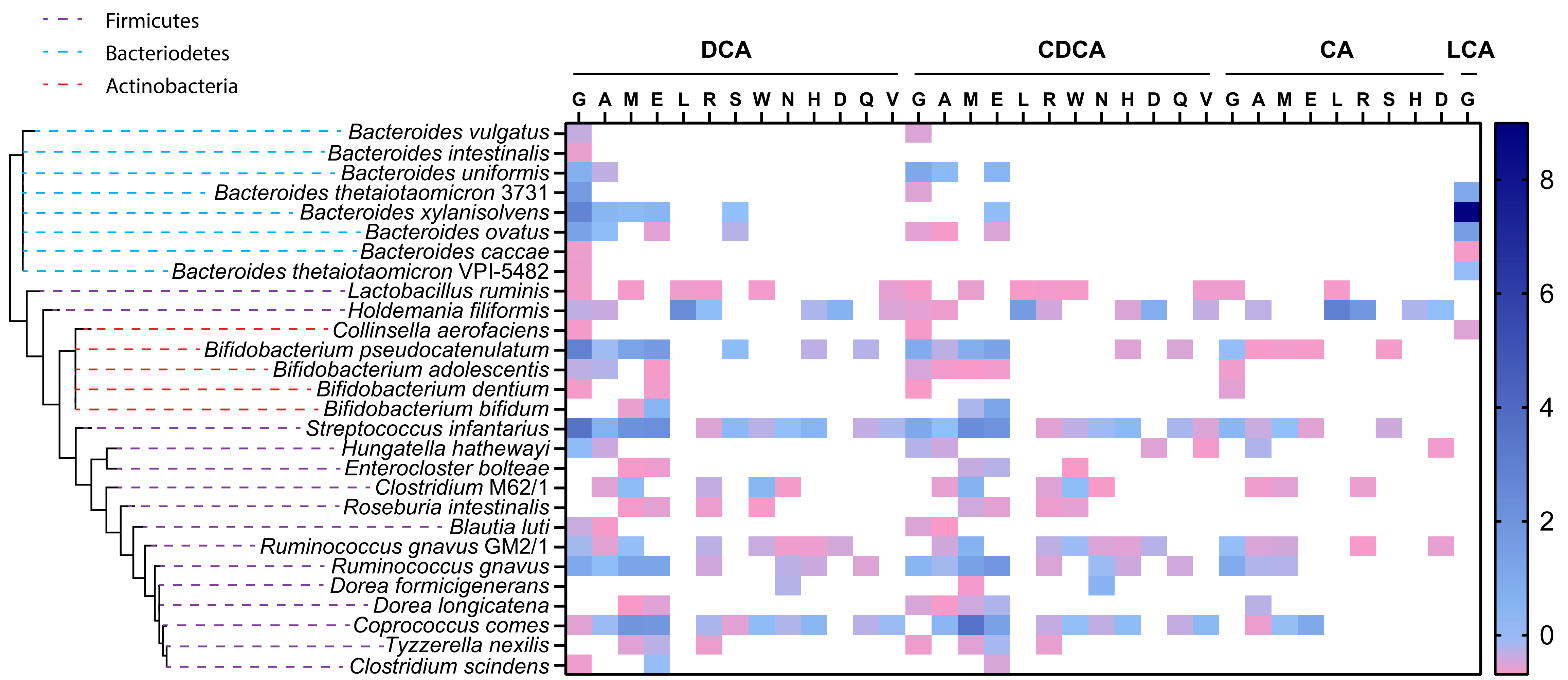

Firmicutes
Bacteriodetes
Actinobacteria
DCA
CDCA
CA
LCA
G A M E L R S W N H D Q V G A M E L R W N H D Q V G A M E L R S H D G
Bacteroides vulgatus
Bacteroides intestinalis
Bacteroides uniformis
Bacteroides thetaiotaomicron 3731
Bacteroides xylanisolvens
Bacteroides ovatus
Bacteroides caccae
Bacteroides thetaiotaomicron VPI-5482
Lactobacillus ruminis
Holdemania filiformis
Collinsella aerofaciens
Bifidobacterium pseudocatenulatum
Bifidobacterium adolescentis
Bifidobacterium dentium
Bifidobacterium bifidum
Streptococcus infantarius
Hungatella hathewayi
Enterocloster bolteae
Clostridium M62/1
Roseburia intestinalis
Blautia luti
Ruminococcus gnavus GM2/1
Ruminococcus gnavus
Dorea formicigenerans
Dorea longicatena
Coprococcus comes
Tyzzerella nexilis
Clostridium scindens
8
6
4
2
0
